# The nucleoplasmic phase of pre-40S formation prior to nuclear export

**DOI:** 10.1093/nar/gkac961

**Published:** 2022-11-02

**Authors:** Jingdong Cheng, Benjamin Lau, Matthias Thoms, Michael Ameismeier, Otto Berninghausen, Ed Hurt, Roland Beckmann

**Affiliations:** Gene Center and Department of Biochemistry, University of Munich LMU, Feodor-Lynen-Str. 25, 81377 Munich, Germany; Institutes of Biomedical Sciences, Shanghai Key Laboratory of Medical Epigenetics, International Co-laboratory of Medical Epigenetics and Metabolism (Ministry of Science and Technology), Fudan University, Dong’an Road 131, 200032 Shanghai, China; BZH, University of Heidelberg, Im Neuenheimer Feld 328, 69120 Heidelberg, Germany; Gene Center and Department of Biochemistry, University of Munich LMU, Feodor-Lynen-Str. 25, 81377 Munich, Germany; Gene Center and Department of Biochemistry, University of Munich LMU, Feodor-Lynen-Str. 25, 81377 Munich, Germany; Gene Center and Department of Biochemistry, University of Munich LMU, Feodor-Lynen-Str. 25, 81377 Munich, Germany; BZH, University of Heidelberg, Im Neuenheimer Feld 328, 69120 Heidelberg, Germany; Gene Center and Department of Biochemistry, University of Munich LMU, Feodor-Lynen-Str. 25, 81377 Munich, Germany

## Abstract

Biogenesis of the small ribosomal subunit in eukaryotes starts in the nucleolus with the formation of a 90S precursor and ends in the cytoplasm. Here, we elucidate the enigmatic structural transitions of assembly intermediates from human and yeast cells during the nucleoplasmic maturation phase. After dissociation of all 90S factors, the 40S body adopts a close-to-mature conformation, whereas the 3' major domain, later forming the 40S head, remains entirely immature. A first coordination is facilitated by the assembly factors TSR1 and BUD23–TRMT112, followed by re-positioning of RRP12 that is already recruited early to the 90S for further head rearrangements. Eventually, the uS2 cluster, CK1 (Hrr25 in yeast) and the export factor SLX9 associate with the pre-40S to provide export competence. These exemplary findings reveal the evolutionary conserved mechanism of how yeast and humans assemble the 40S ribosomal subunit, but reveal also a few minor differences.

## INTRODUCTION

In eukaryotes, ribosome assembly starts in the nucleolus, continues in the nucleoplasm, and is completed in the cytoplasm. In the yeast nucleolus, the formation of pre-ribosomal particles is assisted by hundreds of assembly factors (AFs), which facilitate cotranscriptional folding and modification of the RNA polymerase I-transcribed 35S primary rRNA and the incorporation of ribosomal proteins. Following endonucleolytic cleavage at sites A_2_ or A_3_, which separates the small (18S rRNA) from the large subunit rRNA (5.8S and 25S rRNA), ribosome biogenesis proceeds along separate 40S and 60S ribosome assembly pathways (1–4). In the 60S pathway, domains I, II and VI of the 25S rRNA first assemble into an early pre-60S ribosome, followed later by incorporation of the 5S ribonucleoprotein complex (5S RNP) (1–3,5–7). When these pre-60S particles move from the nucleolus into the nucleoplasm, the Rix1–Rea1 complex checks their assembly state and can trigger further maturation, thereby preparing the pre-60S ribosome for nuclear export into the cytoplasm, where maturation is completed (1–3,8–10).

Maturation of the pre-40S ribosome follows the same principle. Previous studies in fungus and in human cells have revealed the structural transitions underlying these processes in the nucleolus and cytoplasm. In the nucleolus, cotranscriptional folding of 18S rRNA results in the formation of the large 90S pre-ribosome precursor following the same ‘end-to-middle’ order observed for 25S rRNA (11). In detail, the 5'-ETS rRNA, together with the 3' major and minor domains of the 18S rRNA first assemble to form a first transient state of the 90S pre-ribosome (state a). Eventually, with the help of the Kre33 (Kre33, Enp2, Lcp5 and Bfr2) and Krr1 (Krr1 and Faf1) modules, the 5' and central domains of the 18S rRNA can integrate with this preliminary 90S to reach the pre-A_1_ state (11). In this state, the 90S pre-ribosome can recruit the RNA helicase Dhr1 and the Mtr4–exosome RNA nuclease complex to form the small subunit processome (12,13). Driven by the remodeling by Dhr1 and the exosome complex, the 90S pre-ribosome undergoes a shedding process that gradually reduces its size through the dissociation of 90S AFs (14,15). After this 90S to pre-40S transition, a primordial pre-40S ribosome (state Dis-C) emerges with only a handful of 90S AFs still bound, including the U3 snoRNA, Dhr1, the Noc4 module (Noc4, Nop14/Utp2, Enp1, Rrp12 and Emg1), the Bms1 module (Bms1 and Rcl1) and the Mpp10 module (Mpp10 and Imp4), which need to be removed in the nucleoplasmic phase (11,14). In the cytoplasm, the pre-40S mainly goes though quality control processes carried out by the atypical kinases RIOK2 and RIOK1 (Rio2 and Rio1 in yeast), which are responsible for the maturation of the mRNA-binding tunnel of the 40S ribosome. The final maturation steps coincide with the dissociation of RIOK1, which in turn triggers site 3 cleavage by the endonuclease NOB1 (in human cells; Nob1 in yeast), the final act of rRNA trimming (16–22). At earlier states, however, additional AFs are active during the maturation of the pre-40S ribosome, such as BUD23/ TRMT112 (Bud23/Trm112), DIMT1 (Dim1), TSR1, UTP14 and CK1 (Hrr25). BUD23/TRMT112 and DIMT1 are RNA methyltransferases, which methylate G1639 on the 40S head and A1850/A1851 on the rRNA helix 45, respectively (23,24). BUD23/TRMT112 mainly function in the nucleoplasm after replacing Emg1, which in turn could trigger the release of the remaining 90S AFs on state Dis-C (24,25). In contrast, DIMT1 is an even earlier AF which associates already with the 90S pre-ribosome in the nucleolus. In the human cell, DIMT1 is clearly a nuclear protein only functioning in the nucleolar and nucleoplasmic phases (24), while its homologe Dim1 is a more widely distributed factor functioning from the nucleolar until the late cytoplasmic phase in yeast (14,26,27). TSR1 is an inactive GTPase that belongs to the BMS1 GTPase family and binds to the same interface as BMS1. It therefore associates and is active after BMS1 in the nucleus and remains bound to the pre-40S until late maturation phases in the cytoplasm (28,29). UTP14 is a exclusive nucleolar protein that stimulates the RNA helicase activity of DHR1, which drives the transition from the 90S to pre-40S ribosome (30–32). CK1 is a protein kinase that can phosphorylate BYSL and LTV1, thus is thought to trigger the release of the late cytoplasmic AFs, such as BYSL, LTV1, RRP12, PNO1, RIOK2, NOB1 (33–35).

The major development that takes place in the nucleoplasm is dissociation of all 90S AFs and replacement with pre-40S AFs in order to reach an export-competent state that allows the pre-40S ribosome to translocate into the cytoplasm via the nuclear pore complex. In yeast, the exportin Crm1 is necessary for nuclear export of the pre-40S ribosome (36,37). Crm1 interacts, albeit weakly, with the nuclear export signals of Rio2 and Ltv1, indicating that they are essential if the pre-40S is to acquire export competence (38–40). However, to reach sufficient export activity, the additional export adapter proteins Slx9 and Rrp12 are also required (41–44). Slx9 forms a ternary complex with Rio2 and RanGTP, facilitating the recruitment of Crm1 to Rio2 (43). Similarly, Rrp12 can directly interact with the Ran GTPase, but also with nucleoporin FG-repeats (44). In contrast to the well-studied nucleoplasmic maturation of the pre-60S ribosome, the nucleoplasmic processes by which the pre-40S ribosome matures have remained elusive.

Here, using the yeast AFs Slx9 and Dim1 as baits for split-tag affinity purification, and in independent assays using the human AFs SLX9 (also known as C21ORF70), RIOK1, and BYSL (yeast Enp1) for single Flag-tag affinity purification, we isolated nucleoplasmic pre-40S particles, which we investigated by single-particle cryo-electron microscopy (cryo-EM) analysis. By combining yeast and human structures, we uncovered 16 different pre-40S assembly intermediates, most of which represent the long-sought nucleoplasmic pre-40S ribosome. Our results provide a structural inventory and a understanding in principle of the sequential events taking place in the nucleoplasm that underly this decisive pre-40S maturation phase.

## MATERIALS AND METHODS

### Yeast strains

The strains of *Saccharomyces cerevisiae* used in this study are listed in Supplementary Table S1. Genomic tagging was performed as described previously (45). All strains are derived from W303 (46), except the reporter strain PJ69-4A, which was used for the yeast two-hybrid analysis (47).

### Bacterial strains

For plasmid construction, the *E. coli* DH5a strain (Thermo Fisher Scientific) was used.

### Split-tag tandem affinity purification from yeast cells

Yeast strains for single-bait or split-tag tandem affinity purification were harvested during phases of logarithmic growth. Cells were mechanically lysed by using a cryogenic cell mill (Retsch MM400) in lysis buffer containing 50 mM Tris–HCl (pH 7.5), 150 mM NaCl, 2 mM MgCl_2_, 5% glycerol, 0.1% NP-40 and 0.1 mM DTT. The cell lysate was cleared by centrifugation and the supernatant was transferred to IgG Sepharose 6 Fast Flow beads (GE Healthcare) for 2 h at 4°C. Beads were washed with lysis buffer, then subjected to TEV cleavage for 45 min at 16°C. The eluate was transferred to Flag-agarose beads (Anti-Flag M2 Affinity Gel, Sigma–Aldrich) and incubated for 1 h at 4°C. Beads were washed and eluted with buffer containing Flag peptide; elution buffer for cryo-EM analysis contained 50 mM Tris-HCl (pH 7.5), 150 mM NaCl, 2 mM MgCl_2_, 2% glycerol, 0.01% NP-40 and 0.1 mM DTT. Eluted samples were analyzed by SDS-PAGE and stained with colloidal Coomassie (Roti-Blue, Roth).

### Thermophilic Rrp12–Slx9 binding assay

Reconstitution of the thermophilic Rrp12–Slx9 heterodimer was performed as described previously (48). In brief, a preculture of SDC medium was transferred into YPG medium to express *Chaetomium thermophilum* Slx9-ProtA-TEV and Rrp12-Flag gene constructs derived from plasmids under the control of a *GAL1-10* promoter. Cells were harvested after incubation for 6 h and purified by tandem affinity purification as described above.

### Yeast two-hybrid assays

Yeast two-hybrid analysis of *Chaetomium thermophilum* proteins was performed as described previously (48). The yeast strain PJ69-4A was transformed with the plasmids pGADT7 (GAL4 activation domain) and pGBKT7 (GAL4 DNA-binding domain) carrying the respective thermophilic gene. Strains were monitored on SDC−Trp−Leu−His containing 2 mM 3-aminotriazole (3AT) for identification of positive interactions.

### Mass spectrometry

Labeled protein bands in the Coomassie-stained gels were excised and identified by in-house MALDI-TOF mass spectrometry.

### Human SLX9, BYSL, and RIOK1 cell lines

Human SLX9, BYSL, and RIOK1 cell lines were generated as described previously (17,18). In brief, the coding sequences were amplified from a cDNA library. In the case of SLX9, the SLX9 gene was inserted into a modified pcDNA5/FRT/TO plasmid (Invitrogen) containing an N-terminal Strep–Flag tag followed by a 3C cleavage site (pcDNA5-N-TAP-SLX9). In the case of BYSL, the BYSL gene was inserted into a separate modified pcDNA5/FRT/TO plasmid containing a 3C-Flag-Strep at the C terminus (pcDNA5-C-TAP-BYSL). In the case of RIOK1, first, a mutation (D324A) was introduced using site-directed mutagenesis by PCR. Then, an internal Strep–Flag tag following amino acid 496 was added before insertion into the original pcDNA5/FRT/TO vector. To generate the stable cell lines, HEK Flp-In 293 T-Rex cells (Thermo Fisher, R78007) were split one day before into a 10 cm dish and grown to 50% confluence. A mixture of 0.5 μg of the plasmid, 4.5 μg of the helper pOG44 plasmid and 20 μg of polyethylenimine (PEI) was incubated for 25 min then directly added into the culture. Selection was performed with 200 μg/ml hygromycin B (Thermo Fisher) supplied in normal DMEM medium.

### Purification of human pre-40S ribosomes

The human pre-ribosomal complexes were purified as previously described (17,18). In brief, cells in a total of 50 15-cm dishes were cultured to approximately 80% confluence before protein expression was induced for 24 h with 1.6 μg/ml tetracycline. Cells were collected by direct scraping down and washed once in cold phosphate-buffered saline (PBS). Cells were then lysed in lysis buffer [20 mM HEPES (pH 7.4), 150 mM potassium acetate, 5 mM MgCl_2_, 1 mM DTT, 0.5 mM NaF, 0.1 mM Na_3_VO_4_, 0.5% NP-40, 1× protease inhibitor (Sigma–Aldrich)] for 30 min on ice, followed by two strokes using a Dounce homogenizer. After clearing by centrifugation at 10 000g for 15 min at 4°C, the supernatant of the cell lysate was incubated with 100 μl of equilibrated anti-Flag affinity beads (Sigma–Aldrich) for 2 h at 4°C. The beads were then washed four times with 2 ml of wash buffer [20 mM HEPES (pH 7.4), 150 mM potassium acetate, 5 mM MgCl_2_, 1 mM DTT, 0.5 mM NaF, 0.1 mM Na_3_VO_4_, 0.05% NP-40], before the bound material was eluted six times by incubation with 100 μl of a solution containing 20 mM HEPES (pH 7.4), 150 mM potassium acetate, 5 mM MgCl_2_, 1 mM DTT, 0.05% Nikkol and 0.2 mg/ml 3× Flag peptide (Sigma–Aldrich) for 30 min. Finally, the combined eluates were concentrated using 50-kDa molecular mass cut-off filters. The concentration was estimated using a NanoDrop photometer (Thermo Scientific).

### Electron microscopy and image processing

Purified samples (3.5 μl) were applied to pre-coated (2 nm) R3/3 holey-carbon-supported copper grids (Quantifoil), blotted for 2–3 s at 4°C, and plunge-frozen in liquid ethane using an FEI Vitrobot Mark IV. Data were collected on a Titan Krios cryo-electron microscope operating at 300 keV. All data were collected with a pixel size of 1.059 AÅ/pixel and within a defocus range of −0.8 to −2.5 μm using a K2 Summit direct electron detector under low-dose conditions, with a total dose of 44 e^−^/Å^2^. Original image stacks were dose-weighted, aligned, summed, and drift-corrected using MotionCor2 (49). Contrast-transfer function (CTF) parameters and resolutions were estimated for each micrograph using CTFFIND4 and GCTF, respectively (50,51). Micrographs with an estimated resolution of less than 5 Å and an astigmatism of <5% were manually screened for contamination or carbon rupture.

For the human SLX9 sample, a total of 1 768 081 particles were picked from 9379 high quality micrographs. For the human BYSL sample, 1 659 781 particles were picked from 6636 good micrographs. For the yeast Slx9-Dim1 sample, 1 358 874 particles were picked from 7174 good micrographs. Subsequently, reference-free 2D classification in Relion 3.1 was used to exclude contaminating particles (52), which resulted in 1 389 754 particles for the SLX9 sample, 212 733 particles for the BYSL sample, and 1 292 470 particles for the Slx9-Dim1 sample. Focused classification and general overall classification were both applied to sort the different states in Relion, as outlined in Supplementary Figure S2 (52). Particles representing the desired states were refined to high resolution in Relion (52). In order to facilitate model building, multibody refinement was carried out to improve the local resolution for the human RRP12 states. In general, the pre-40S ribosome was divided into two bodies: the 40S ribosome body region and the 40S ribosome head region. All the final maps were post-processed and local-resolution-filtered using Relion (52).

The purification and image processing of the human RIOK1 sample has been reported previously (17). Briefly, 7712 movies were collected with a pixel size of 1.059 Å and defocus of 0.5–2.5 μm using a K2 Summit direct electron detector under low-dose conditions (48 frames at approximately 1 e^−^ Å^−2^) using EPU (FEI Company). Gain-corrected movie frames were motion corrected and summed with MotionCor2(49) and contrast-transfer-function (CTF) parameters were determined with CTFFIND4(51) and Gctf(50). After manual screening for image quality, 7365 micrographs were used for automated particle picking by Gautomatch (https://www2.mrc-lmb.cam.ac.uk/download/gautomatch-056/). Totals of 1 822 874 particle images were then sorted using reference-free classification and 3D refinement in Relion 3.0(52). After sorting, particles from each state were separately subjected to Bayesian polishing, CTF refinement and 3D refinement in Relion(52) to get the final reconstruction. To better visualize the 40S Head region, multibody refinement was carried out with two bodies (40S Head and Body region). The final map was post-processed and local-resolution-filtered using Relion (52).

### Model building and refinement

In general, structures of the yeast cytoplasmic pre-40S ribosome (PDB IDs: 6ZQG and 6EML) (14,19) and the human pre-40S ribosome (PDB IDs: 6G18 and 6G51) were used as initial references to generate homology models or rigid-body fits (18), followed by manual adjustment in Coot (53). Because certain subregions were not well resolved, mixed full models (polyalanine and sidechain based) were prepared. In general, if a protein, or a part of one, is represented as polyalanine, the resolution of the corresponding map did not allow a *bona fide* molecular model to be built.

For the state Tsr1, the yeast model (PDB IDs: 6ZQG and 6EML) were rigid-body fit into the density in Coot. Due to the low local resolution, the 40S head part was excluded from the final model. The N terminus of Tsr1 was manually built in Coot, as was the N-terminal helix situated under h44 of the 18S rRNA. The conformation of the rest of the global domain in state Tsr1-3 was taken from the yeast model (PDB ID: 6EML) (19). The same model also was used for the state Tsr1-2, however, manual adjustments were made to fit the flip-out conformation. For the sub-states that have uS5 incorporated, the yeast uS5 was rigid-body fit.

For the state RRP12, the human model (PDB ID: 6G18) (18) was rigid-body fit into the density. Compared to the model, the conformation of the 40S head is completely different, and thus manual adjustment was carried out in Coot to fit the density. Helices h18 and h34–h40 of the 18S rRNA were built *de novo* in Coot once high-resolution density was available. The AFs RRP12, ENP1, LTV1, TSR1, BUD23, TRMT112 and SLX9 were of sufficient resolution, thus models were built *de novo*. The models for the AFs PNO1, RIOK2 and NOB1 were also taken from the human model (PDB ID: 6G18) (18), although they also had high resolution. Because of the low local resolution, the crystal structure of human CK1 delta (PDB ID: 6GZM) was rigid-body fit into the CK1 density without further adjustment (54). The yeast state Rrp12-A had insufficient resolution to build a model.

For the human state UTP14, the model of the human state RRP12-A1 was used as a starting point; the flexible 40S head was manually removed from the final model. The crystal structure of the human DIMT1 (PDB ID: 1ZQ9) was rigid-body fit into the DIMT1 density. The human UTP14 was built *de novo* in Coot.

For the human state RIOK2-D2, a human model (PDB ID: 6G51) (18) was used. TSR1 was manually removed since it had already dissociated from the particle. For the yeast state Rio2-C, the available model (PDB ID: 6EML) (19) was used. The Dim1 structure from PDB ID 6ZQG (14) was used to fit the Dim1 density. However, the Nob1 model is a homology model generated using SWISS-MODEL (55) based on the human NOB1 model (PDB ID: 6G18) (18).

With the exception of the Rrp12-A, Rio2-C and RIOK2-D2 states, the final models for the rest of the states were real-space refined with secondary structure restraints using the PHENIX suite (56). Final model evaluation was performed with MolProbity (57). Maps and models were visualized and figures created with ChimeraX (58).

## RESULTS

### Cryo-EM analysis of the eukaryotic nucleoplasmic pre-40S ribosome

**Figure 1.**
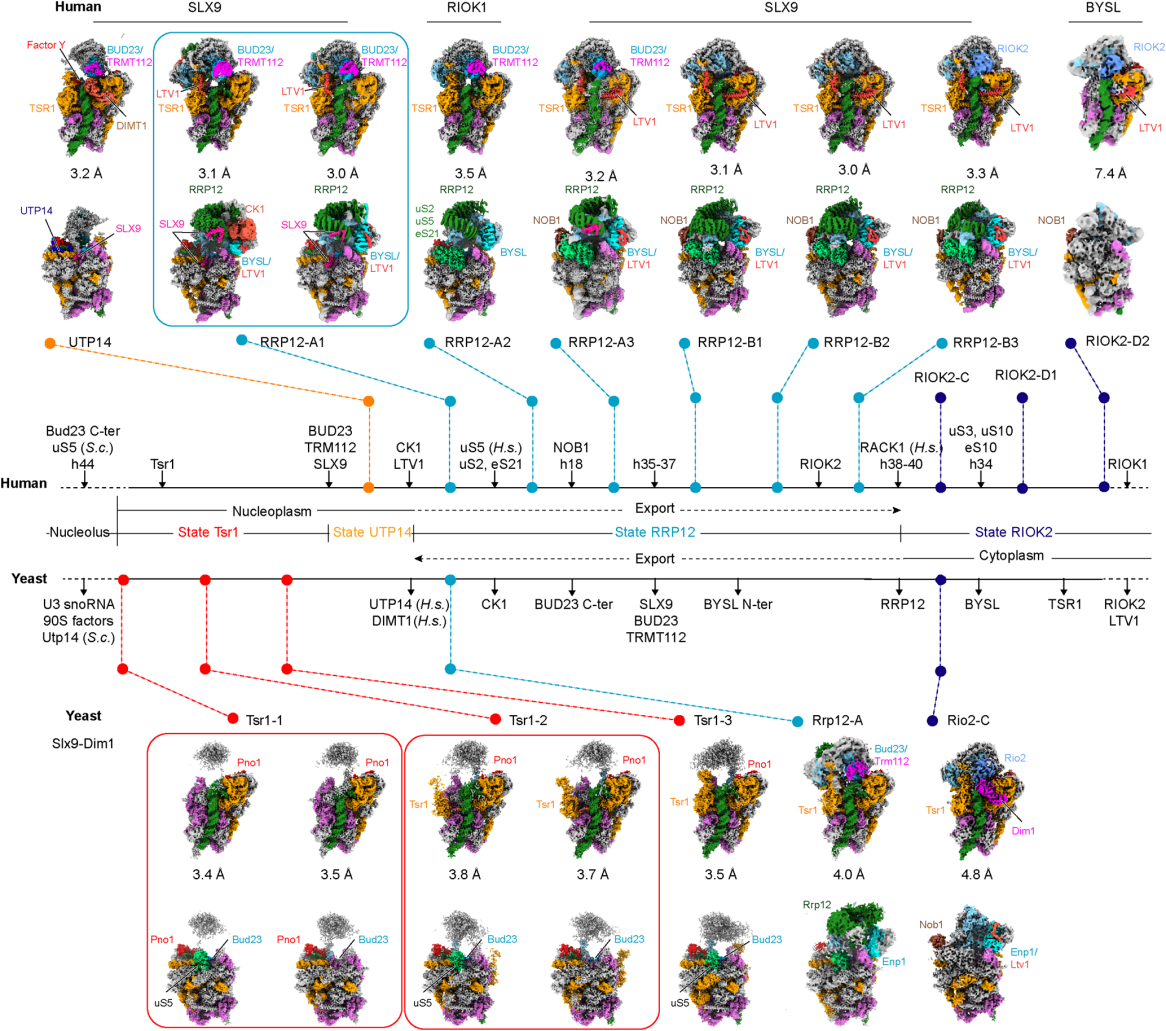
Cryo-EM analysis of nucleoplasmic pre-40S intermediates. Cryo-EM maps of distinct states of human (top) and yeast (bottom) nucleoplasmic pre-40S intermediates observed after affinity purification using SLX9, RIOK1, BYSL (human) and Slx9–Dim1 (yeast). Two views (front and back) of each state are presented, with average resolution expressed in (Å). The central scheme illustrates the compositional changes taking place between each state and the sequence in which they occur. Sub-states for which the assembly order cannot be clearly distinguished are grouped. The bait proteins used for purification are indicated above the respective structures. *S.c.: Saccharomyces cerevisiae, H.s.: Homo sapiens*.

Previous studies revealed the structural basis of how the eukaryotic 40S ribosome biogenesis pathway takes place in the nucleolus and cytoplasm (14,17,18), but the maturation events occurring in the nucleoplasm remained largely unknown. To gain insight into this step, we carried out additional split-tag affinity purifications from yeast cells using two AFs as bait proteins under normal growth conditions (Supplementary Figure S1A). First, we pulled on Slx9, which is a nuclear export adapter for exportin (42,43), and then used Dim1 (DIMT1 in human) as a second bait, which interacts with 90S intermediates but is retained in the later cytoplasmic pre-40S ribosome. In parallel, we also isolated pre-40S particles from human cells, using a single-step purification with SLX9, RIOK1 (D324A), or BYSL expressed as bait proteins in human HEK293 cells displaying wildtype growth (Supplementary Figure S1B). Using cryo-EM analysis we identified 16 distinct intermediates. Notably, earlier nucleoplasmic states were enriched in the pull-downs from yeast and later states were enriched from human cells (Figure 1, Supplementary Figures S2–S4, and Supplementary Tables S2–S4). To our surprise the (cytoplasmic) RIOK1 bait revealed a novel intermediate state that is clearly nucleoplasmic and not RIOK1 associated (state RRP12-A2). Apparently, this preparation yielded, in addition to bona fide RIOK1 containing particles, other non-specifically bound 40S complexes such as 43S pre-initiation complexes (17,59). All observed intermediates can be assigned to three different major phases: (i) the Tsr1 phase, in which after the remaining 90S AFs have dissociated, the association of new nucleoplasmic AFs such as Tsr1 facilitates the first coordination between head and body (states Tsr1 and UTP14; see below for a summary of the notation adopted in this study); (ii) the RRP12 phase, which is characterized by the association of RRP12 and further maturation of the head and (iii) the cytoplasmic/nucleoplasmic RIOK2 phase that follows RRP12 dissociation (state RIOK2) (Figure 1). Based on the increasingly matured state of the pre-40S (ribosomal protein composition/rRNA conformation) and AF composition, it was most plausible to position all intermediates in a sequential order. Yet, we cannot exclude that additional parallel pathways exist with different intermediates, which we did not catch in our purification strategy.

In the yeast Slx9–Dim1 preparation, we observed four previously unknown states (states Tsr1-1, Tsr-2, Tsr1-3 and Rrp12-A1), which reveal the distinct recruitment steps of the Tsr1 protein. Notably, the yeast state Tsr1 (with sub-states 1–3) has lost all remaining 90S AFs. However, all sub-states bear the fully assembled central pseudoknot (CPK), a new premature conformation of the 18S rRNA helix 18 (h18) and each display an almost complete delocalization of the 40S head. The conformation of h18 is stabilized by Bud23, which unambiguously places the Tsr1 states between the primordial pre-40S ribosome (state Dis-C) (14) and the later states RRP12/Rrp12. In contrast to the human intermediates, which display associated uS5 in the RRP12 state, uS5 in yeast can apparently incorporate into the pre-40S ribosome already in earlier states before state Tsr1-3 is reached. However, the time point at which this occurs is not clearly defined; indeed, states Tsr1-1 and Tsr1-2 were both observed with and without uS5 incorporated. Another novel early state, which we term UTP14, was found in the human SLX9 preparation. This intermediate showed complete TSR1 incorporation and density corresponding to DIMT1 and also to UTP14 in the same conformation observed in the yeast primordial pre-40S (Dis-C) (14). Highly similar to the yeast Tsr1 state, the human UTP14 state also displayed an almost completely delocalized 40S head, which appears to be the hallmark of early nucleoplasmic pre-40S intermediates, which arise after the 90S to primordial pre-40S transition (Figure 1).

In addition to state UTP14, in the human preparations, we found seven later nucleoplasmic intermediates. Two of these resemble known assembly states, but could now be sufficiently enriched to be characterized at molecular resolution (18). The known human state RRP12-A1 (previously ‘state A’) was reconstructed to approximately 3 Å, allowing us to build a near complete model specifically including the AFs BUD23, TRMT112, RRP12, LTV1, TSR1 and BYSL. In addition, we identified and built SLX9 and the casein kinase 1 (CK1; Hrr25 in yeast) in these intermediates. The state RRP12-A1 existed with and without CK1 bound, however, the limited local resolution did not allow to distinguish the two CK1 isoforms known to be involved in 40S ribosome assembly (CK1δ and CK1ϵ) (33–35). In parallel, we observed particles in the so-far elusive state Rrp12-A1 at medium resolution in yeast, which indeed correspond to the assembly state RRP12-A1 in human. According to the sequential recruitment of the uS2–uS5–eS21 ribosomal protein cluster and NOB1, we identified two more new sub-states, RRP12-A2 and RRP12-A3, respectively. Similarly, we could provide a complete near-atomic model for human RRP12-B (previously ‘state B’), which could now be classified into three subpopulations - referred to as states B1, B2 and B3-based on the different conformations of RRP12 and the early recruitment of RIOK2, respectively. Unlike the previously published cytoplasmic yeast pre-40S ribosome, our newly identified yeast state Rio2-C associated with Nob1 (together with Dim1) (19–21). Finally, the human BYSL purification yielded two particles, of which one had been previously documented as ‘state D’ (now state RIOK2-D1). The new state, RIOK2-D2, showed complete dissociation of TSR1 (18). The characteristic features of all these later states are their further maturation and, more importantly, integration of the head (3' major) domain into the 40S architecture (Figure 1).

In order to simplify the above used pre-40S particle nomenclature, we defined these states after the main functional factors. Therefore, the old states A and B were renamed to state RRP12-A1 and states RRP12-B1, respectively. State C (yeast and human) was renamed RIOK2-C and Rio2-C in human and yeast, respectively; the human state D became state RIOK2-D1 (18).

### Highly dynamic 40S head conformation after 90S to 40S transition

**Figure 2.**
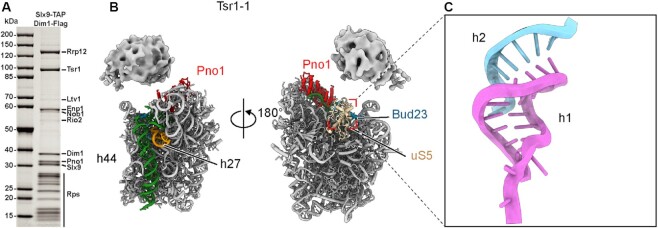
The 40S head is highly flexible in the early nucleoplasmic Tsr1 state particle. (**A**) SDS-PAGE analysis of the yeast pre-40S intermediates purified using Slx9–Dim1 split-tag; ribosome assembly factors identified by mass spectrometry are indicated. (**B**) Two views of the molecular model of the yeast state Tsr1-1 (with uS5). The highly flexible 40S head is indicated using the density map. (**C**) The central pseudoknot (h2 of the 18S rRNA) formed in yeast state Tsr1-1.

We first attempted to isolate nucleoplasmic pre-40S ribosome particles from logarithmically growing yeast cells (Supplementary Figure S1). Based on previous data on nucleolar and cytoplasmic states, the proteins purified using yeast Slx9–Dim1 as split bait exhibited a distinct intermediary profile, in which the early 90S AFs (the Mpp10 and Bms1 modules, Dhr1, Noc4, Nop14 and Emg1) were already lost, but late pre-40S factors (Rio2, Ltv1 and Nob1) were less enriched (27) (Figure 2A). Consistent with the suggestion that Rrp12 and Slx9 act as adapters for nuclear export and with Rio2 being a hallmark of cytoplasmic particles (42,44), this sample provided us the nucleoplasmic pre-40S particles of interest, as shown by single-particle cryo-EM analysis. The most notable feature of Tsr1-1, the earliest state, is the highly flexible 40S head. This observation was not too surprising since the head of the primordial pre-40S (state Dis-C) is highly stretched and mostly dynamic and invisible (14) (Figure 2B and Supplementary Figure S5A, B). It is thus reasonable to speculate that after the dissociation of remaining 90S factors, the flexible 40S head is even more dynamic at first, and primed for new factors to integrate into the pre-40S particle and drive conformational maturation.

Unlike the highly dynamic 40S head, the 5' and central domains of the 18S rRNA are almost mature, as in the primordial pre-40S (14) (Supplementary Figure S5A, B). However, state Tsr1-1 has already lost all its remaining 90S AFs, including the U3 snoRNA. This allows the helices h27 and h44 of the 18S rRNA to mature, and the CPK to fully assemble (Figure 2C and Supplementary Figure S5C, D). Although, we were missing the states that could show the remodeling of the primordial pre-40S (now described by Cheng *et al.NAR*), the Tsr1 states are likely to represent the earliest nucleoplasmic intermediates, in which the 40S head is prepared for further remodeling.

### Stabilization of the pre-40S head precedes nuclear export

**Figure 3.**
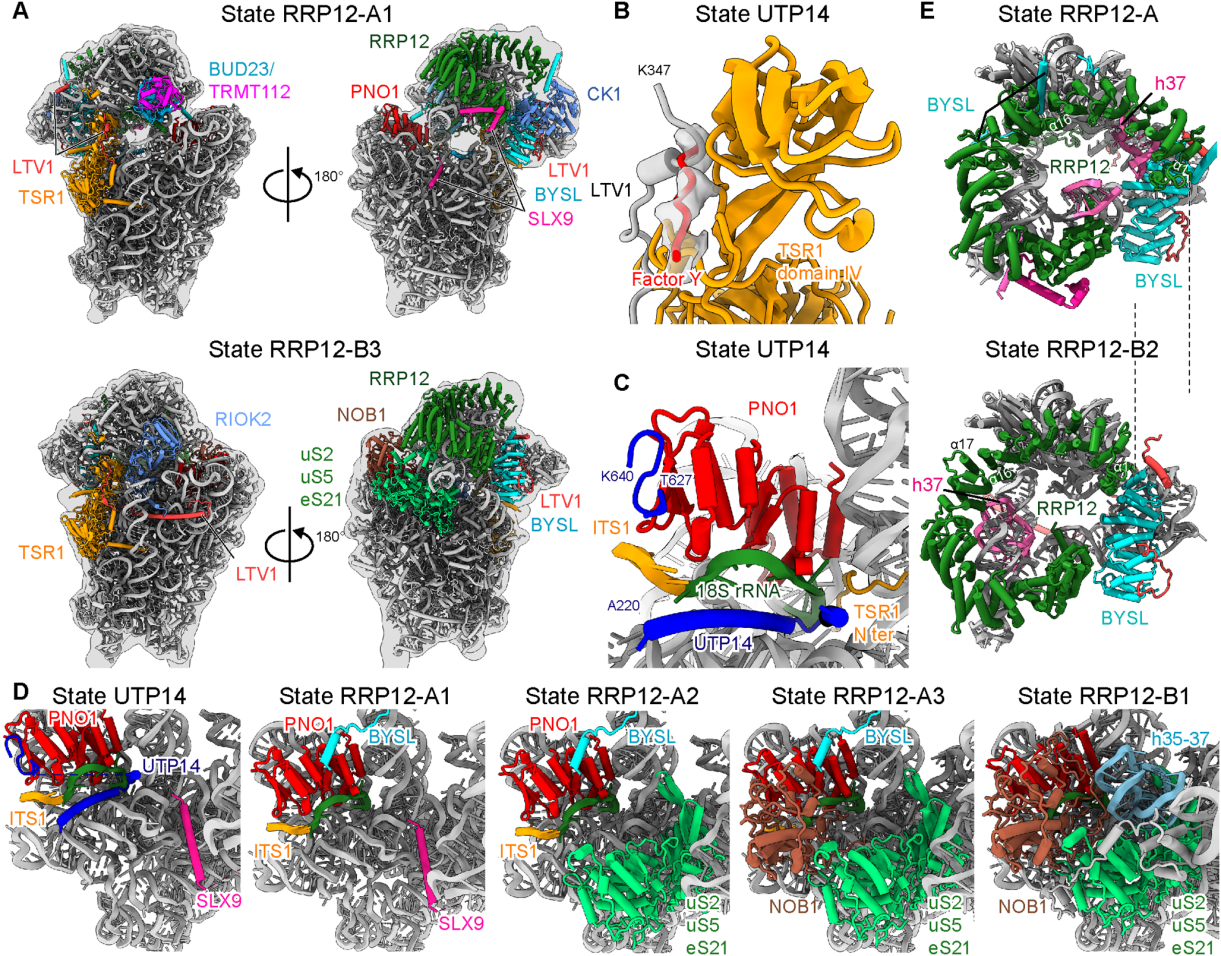
The 40S head is stabilized in the export-competent state RRP12. (**A**) Two views of the molecular models of human states RRP12-A1 and RRP12-B3; all assembly factors are indicated. (**B**) The position at which the middle region of LTV1 interacts with domain IV of TSR1 in state RIOK2-C is occupied by an unidentified factor in the human state UTP14. (**C**) In human state UTP14, UTP14 interacts with PNO1. Together, these two interact with the 3' region of the 18S rRNA and ITS1 rRNA. (**D**) The sequence of maturation events occurring in the CPK and 3' regions of the 18S rRNA (from left to right: states UTP14, RRP12-A1, RRP12-A2, RRP12-A3 and RRP12-B1). (**E**) In the transition from state RRP12-A to RRP12-B2, the N-terminal ARM repeat of RRP12 slides to the one side. The movement of the N terminus is indicated with dash lines.

In contrast to the split-tag purification in yeast, purification using SLX9 as single bait in human cells mainly provided us with later nucleoplasmic intermediates in states UTP14 and RRP12 (Figures 1, 3A, and Supplementary Figure S6A). Like the yeast state Tsr1, state UTP14 also has a highly flexible 40S head, however, it had already undergone the complete recruitment of TSR1, DIMT1, BUD23, and TRMT112 (Figure 1 and Supplementary Figure S6A). Thus, state UTP14 represents a new late nucleoplasmic intermediate following the Tsr1 state observed in yeast. It is highly likely that neither LTV1 nor NOB1 can engage during this phase. First, despite the low resolution of the BYSL region on the 40S head, the binding interface of the C terminus of LTV1 near h45 of the 18S rRNA was clearly occupied by DIMT1, which is exclusively a nuclear protein in human cells (18) (Figure 1 and Supplementary Figure S6A). Second, the binding interface of the middle region of LTV1, next to domain IV of TSR1, was occupied by an unidentified factor (18) (Figure 3B). Last, two of the major binding interfaces of NOB1 on pre-40S (18), one on PNO1 and the other clamping the 3' end of the 18S rRNA, were both masked by UTP14 (Figure 3C). Thus, state UTP14 is not ready for export and has to undergo the RRP12-associated maturation steps.

In contrast to state UTP14, the human state RRP12 is further matured at the 40S head, which has a more rigid conformation and could be resolved to high resolution. In addition to providing a near complete model for AFs BUD23, TRMT112, TSR1, BYSL, NOB1, LTV1 and RRP12, we could newly assign the positions of CK1 and SLX9 in state RRP12-A (Figure 3A). Moreover, we were able to visualize a detailed assembly process, setting up endonuclease NOB1 in its inactive conformation to await the signal from RIOK1 to execute the final cleavage at site 3 (17). This includes recruitment of the uS2–uS5–eS21 cluster and NOB1 and maturation of 18S rRNA helices h35–37 (Figure 3D). We also observed a conformational change in RRP12 in the RRP12-B2 state (Figure 3E). In state RRP12-A, the central region (around α-helices 16 and 17) and one of the C-terminal regions (residues 1100–1135) of RRP12, together with the N-terminal region (residues 1–72) of BYSL, were tightly associated with the h41 region of the 18S rRNA (Supplementary Figure S6B). In this conformation, the N-terminal ARM repeats of RRP12 could stabilize the h35–37 region of the 18S rRNA in its immature position. As maturation progresses to state B1 and helices h35–37 adopt their mature conformation, RRP12 loses its interaction with BYSL and, in transition to state B2, the N-terminal ARM repeats of RRP12 slide outwards, which might be a requirement for its release (Figure 3E and Supplementary Figure S6B).

Several factors have been reported as nuclear export adapters for the pre-40S ribosome, including LTV1, SLX9, RIOK2 and RRP12 (38,39,42,44), which are all present in our RRP12 state. We thus conclude that the observed RRP12 state is the principal nuclear export state, whereas the UTP14 state is a late nucleoplasmic state in humans. If this is the case, the maturation and integration of the 40S head represents the required signal to render the pre-40S intermediate competent for nuclear export.

### Bud23 stabilizes h18 in an immature conformation in the nucleoplasm

**Figure 4.**
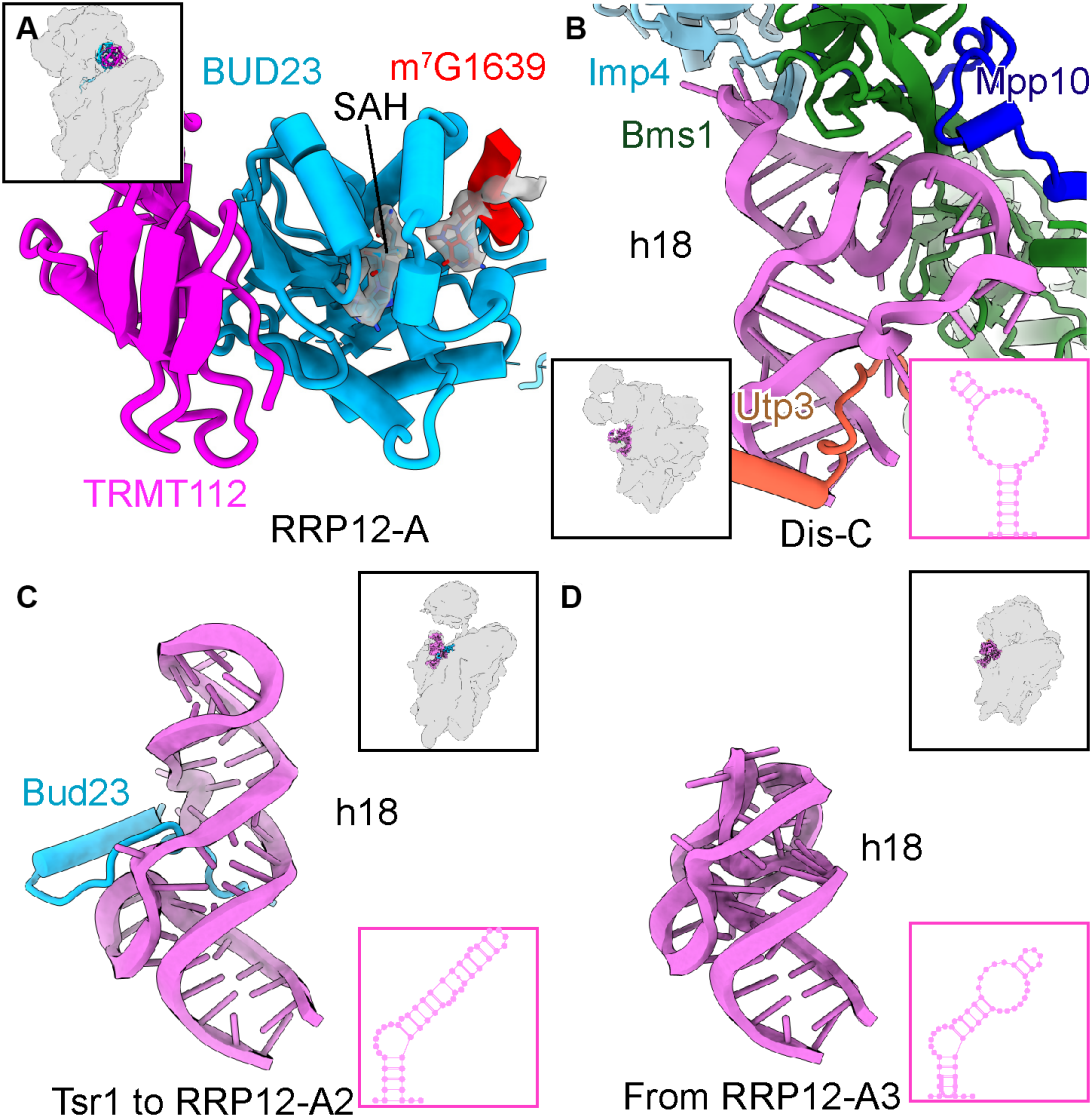
BUD23 maintains h18 of the 18S rRNA in a new immature conformation. (**A**) A molecular model of the complex formed between TRMT112 and the methyltransferase domain of BUD23. SAH and m7G1639 of the 18S rRNA are shown in stick representation surrounded by density map. (B–D) The conformation of h18 of the 18S rRNA in state Dis-C (**B**), Tsr1-1 to RRP12-A (**C**) and RRP12-B1 (**D**). The assembly factors responsible for keeping the 18S rRNA h18 immature are shown. The secondary structures of h18 are shown inset in the lower right corners of each panel.

To reach the export competent state, the activity of several AFs is required, of which BUD23 acts early after the 90S to 40S transition (23). In state RRP12-A, BUD23 binds to the same interface as Emg1 in the yeast primordial pre-40S (14). A recent study revealed that replacement of Emg1 coincides with dissociation of surrounding factors, thus promoting further pre-40S maturation (25). In our structure, BUD23 has high structural similarity (RMSD = 0.775 Å for 177 residues) when comparing with the X-ray structure of the yeast Bud23 bound to SAM (Figure 4A and Supplementary Figure S7A) (60). In human cells, BUD23 has an N-terminal methyltransferase domain, which can methylate G1639 at the N7 position (G1575 in yeast) with the help of its cofactor TRMT112, and a flexible C terminus (23,24). Consistently, in our RRP12-A state, we could clearly identify the base G1639 inside the substrate binding pocket of BUD23 (Figure 4A). Although its activity is not essential for progression of pre-40S (23,24), based on the density map, we assigned the ligand inside the pocket as *S*-adenosyl-L-homocysteine (SAH), with the N7 of G1639 already methylated (Figure 4A and Supplementary Figure S7B).

In addition to the methyltransferase domain, we also built the C terminus of BUD23 starting with the earliest yeast state Tsr1-1, in which it binds to the immature h18 of the 18S rRNA. Compared with the primordial pre-40S in yeast, h18 adopts a new immature conformation, thus being the last part of the 5' domain to complete its folding (Figure 4B, C). The C terminus of BUD23 apparently aids stabilization of this new immature conformation, and only once it is released in state RRP12-B1 can h18 adopt its fully mature conformation forming the mRNA entry tunnel (Figure 4D). Because in the yeast primordial pre-40S, most of the 90S factors are associated with the immature h18 (the Bms1 and Mpp10 modules), this might provide an explanation for their release facilitated by BUD23 binding. Taken together, BUD23 plays an important role in shaping the 40S head because the N-terminal methyltransferase domain of BUD23 binds to the decoding center, connecting the 40S head and body while the C terminus coordinates h18 of the 18S rRNA.

### Complete activity cycle of TSR1 in early head stabilization and maturation

**Figure 5.**
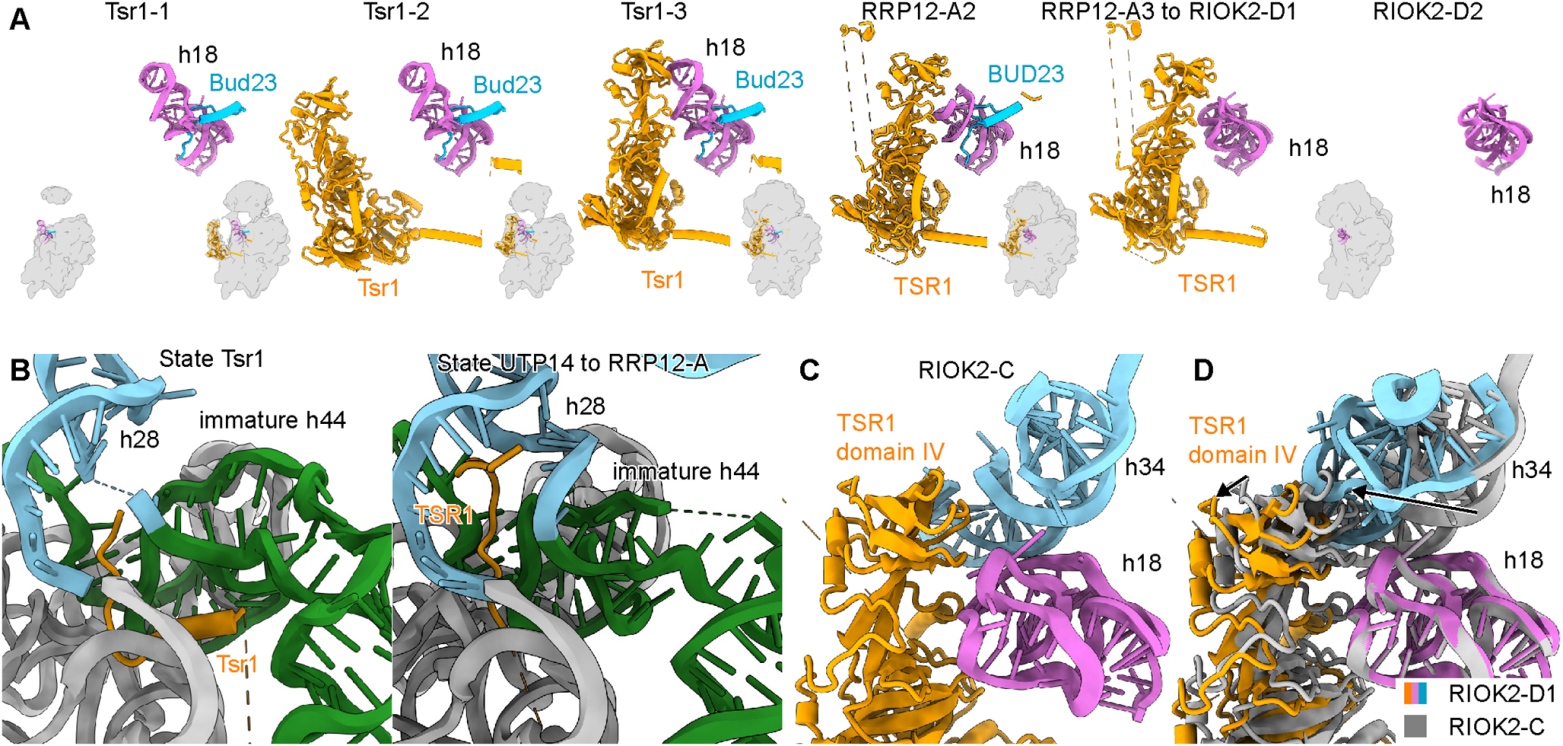
The complete activity cycle of TSR1 in early head maturation. (**A**) The complete activity cycle of TSR1. The immature/mature h18 of the 18S rRNA, Bud23/BUD23 and Tsr1/TSR1 are shown in ribbon representation (left to right: states Tsr1-1, Tsr1-2, Tsr1-3, RRP12-A, RRP12-B1 to RIOK2-D1 and RIOK2-D2). The overall positions are shown in thumbnails. (**B**) The very N terminus of the yeast Tsr1 (left, state Tsr1) and human TSR1 (right, from state UTP14 to RRP12-A) interferes with the maturation of h28 of the 18S rRNA. (**C, D**) The conformation of domain IV of TSR1 in states RIOK2-C and RIOK2-D1. Maturation of h34 of the 18S rRNA pushes domain IV of TSR1 outward. The movements of h34 and domain IV of TSR1 are indicated by arrows.

Similar to BUD23, TSR1 is another AF that connects the head and body of the pre-40S subunit, thereby contributing to the progress of h18 rRNA maturation and head-to-body positioning/coordination. By combining the different intermediates from both yeast and human, we can visualize a near complete picture of the cycle of TSR1 activity on the pre-40S ribosome. This cycle accompanies the progress from an almost completely flexible immature head in the nucleoplasm to a fully matured 40S head in the cytosol (Figure 5A). After the release of Bms1 and association of Bud23, the Tsr1-1 state in yeast, which directly precedes Tsr1 recruitment, displays the rRNA h18 in a new immature conformation and a highly flexible 40S head. This state is followed by state Tsr1-2, in which Tsr1 is recruited by insertion of its N terminus between the immature rRNA helices h28 and h44–45 (both yeast and human) and its N-terminal helix between h44 and the 5' domain of the 18S rRNA (Figure 5A and Supplementary Figure S7C). This is consistent with our biochemical data showing that the N-terminal Tsr1 fragment alone (1–84, also carrying the endogenous Tsr1 NLS) can incorporate into the pre-40S particle. One possibility is that this fragment competes directly with the binding of full-length Tsr1 protein (Supplementary Figure S7D, E). Although the very N terminus of Tsr1 adopts slightly different conformations in human and yeast complexes, in both organisms it binds to the same conserved interface, that is, next to rRNA h44–45 (Figure 5B). In this position, it reaches deep into the h28 of the 18S rRNA, thus it might either sense or stabilize the immature folding of h28 and prevent complete formation of the 40S neck until (human) state RRP12-B1 is reached (Figure 5B). In this later state, the far N terminus of TSR1 was invisible and h28 of the 18S rRNA was already mature. In addition to the N terminus, in state Tsr1-2, the C-terminal domains of Tsr1 are in a flipped-out conformation, leading to the positioning of domain IV of Tsr1 at a substantial distance from h18 of the 18S rRNA and therefore unable to interact (Figure 5A). A similar flipped-out conformation has been recently observed both in human and yeast (61,62), when TSR1 binds to fully matured 40S or 80S-like ribosomal complexes, respectively. Eventually, from yeast state Tsr1-3 onward, domain IV of Tsr1 interacts with immature h18 of the 18S rRNA. However, h18 will be fully matured only later with domain IV of TSR1 still bound between states RRP12-B1 and RIOK2-D1, indicating that Tsr1 should contribute to the maturation of this helix (Figure 5A). We observed the state RIOK2-D2 as the first state after Tsr1 dissociates, although we have no clear indication of how the release of Tsr1 is triggered. Notably, in state RIOK2-D1, incorporation of the uS3 ribosomal protein cluster and the maturation of h34 of the 18S rRNA pushes domain IV of TSR1 outwards (Figure 5A, C). It is therefore plausible to speculate that dissociation of TSR1 is triggered by this further matured structural arrangement, and that it might resemble the reverse recruitment pathway.

Taken together, Tsr1 appears to be the earliest nucleoplasmic AF to spatially coordinate the immature head with the body of the pre-40S. It maintains this scaffolding function for head formation from an early nucleoplasmic phase all the way through the export and to the cytoplasmic phase of 40S maturation.

### RRP12 serves as a hub for coordinating head maturation

**Figure 6.**
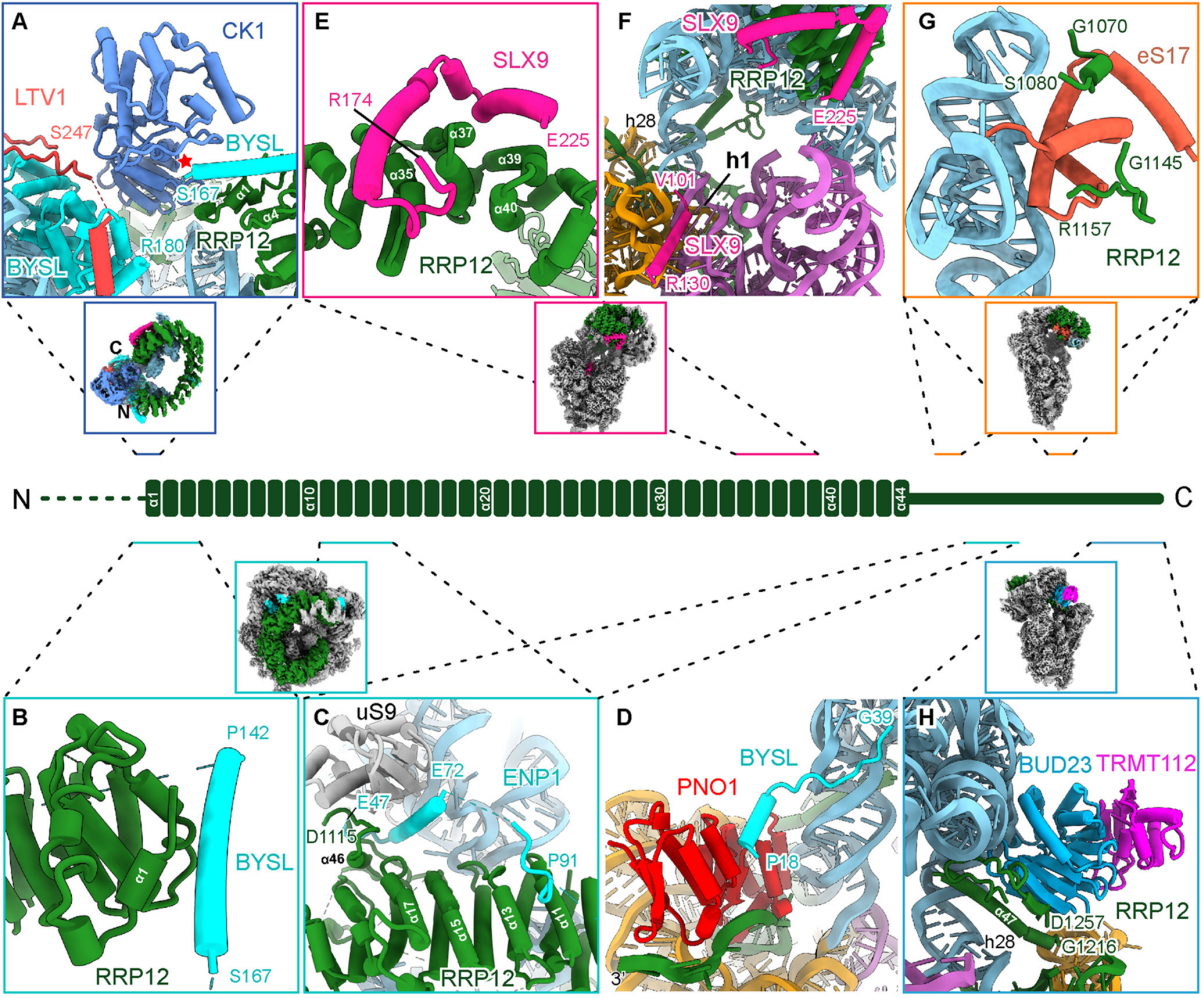
RRP12 is the central coordinator for head formation. (**A**) The N and C termini of RRP12 coordinate CK1 and position it close to BYSL and LTV1. The active center of the kinase domain is indicated with a red star. (**B**) The N-terminal ARM repeat of RRP12 interacts with the 142–167 region of BYSL. (**C**) The middle region of the ARM repeat of RRP12 interacts with the 47–91 region of BYSL. (**D**) The N terminus of BYSL (residues 18–39) interacts with PNO1. (**E**) The region of amino acids 174–225 of SLX9 interacts with the C-terminal ARM repeats of RRP12. (**F**) The region of amino acids 101–130 of SLX9 binds to the CPK region on the 40S body. (**G**) Two regions of RRP12 (residues 1070–1080 and 1145–1157) interact with eS17. (**H**) The very C terminus of RRP12 pokes through the h28 region and interacts with BUD23. Center: A schematic representation of the architecture of RRP12; thumbnails indicate the overall position of the respective assembly factor.

Rrp12 is an AF that in yeast is recruited to the early 90S particle as a component of the Noc4 module (Noc4, Nop14, Emg1, Enp1, Rrp12) (14,63,64). RRP12 is a large ARM-repeat protein and consists of 44 protein helices in the center and two long N-terminal and C-terminal extensions (Figure 6). It is primarily associated with helices h34–40 of the 18S rRNA, thus keeping this region far away from its mature position both in the 90S and later nucleoplasmic pre-40S particles (14). rRNA helices h35–37 already adopt their mature position in human state RRP12-B1, whereas helices h38–40 can only fully mature after RRP12 is released from the pre-40S in state RIOK2.

The ARM-repeat region of RRP12 forms an almost closed circle, with its N-terminal and C-terminal repeats providing an interface to bind CK1, as observed in the RRP12-A1 state (Figure 6A). Although CK1 is not required for nuclear export of pre-40S particles, it can phosphorylate BYSL and LTV1, thereby triggering their release from the pre-40S particle (22,33,34). In the RRP12-A1 state, CK1 is already well positioned in the vicinity of both LTV1 and BYSL, however, its kinase-active center faces away from the BYSL–LTV1 complex (Figure 6A). This indicates that CK1 is recruited to the pre-40S in the early state RRP12-A, but remains in an inactive state. Although we did not observe density for CK1 in subsequent states, it might still flexibly associate with the pre-40S in the RRP12-B and RIOK2 states. During maturation in the cytoplasm, CK1 could phosphorylate BYSL and LTV1 promoting their release and recycling. This scenario is consistent with previous studies showing that CK1 can be co-purified both with early (RRP12, BYSL, SLX9, PNO1) and late (LTV1, RIOK2) pre-40S AFs. However, phosphorylation only occurs in the late cytoplasmic particles (33).

Besides its rRNA and CK1 interaction, Rrp12 has been shown to directly interact with Enp1 in the Noc4 module. Using yeast two-hybrid analysis, we mapped the interface to the ARM-repeat region of Rrp12 (Supplementary Figure S8A, B) and the N terminus of Enp1 (Supplementary Figure S8A, C). Consistent with this observation in yeast, we found in human state RRP12-A that the N terminus of BYSL binds to the ARM repeat of RRP12 in three different regions. First, residues 142–167 of BYSL form an α-helix that closely interacts with the beginning of the ARM repeat of RRP12 (Figure 6B). Second, a loop (residues 83–91) of BYSL is situated atop helices 11 and 13 of RRP12 (Figure 6C). Third, helices 47–72 of BYSL poke through a tunnel formed by helices 15–17 and the C-terminal region (residues 1102–1135) of RRP12, uS9, and h41 of the 18S rRNA (Figure 6C and Supplementary Figure S6B). These interactions appear to be important for the stable association of RRP12 to the pre-40S, since loss of these interactions, starting in state RRP12-B2, allows the subsequent release of RRP12. Interestingly, the very N terminus of BYSL reaches along h28 of the 18S rRNA to bind between the two KH domains of PNO1, the same surface of PNO1 that later interacts with NOB1 in state RRP12-B1, thereby preventing premature incorporation of NOB1 (Figure 6D).

Another RRP12 interactor is the AF SLX9. Although we were using SLX9 as bait for affinity purification in both human and yeast samples, we only observed it in the human states UTP14 and RRP12-A. We discovered that a C-terminal region (residues 174–225) of SLX9 formed two helices that pack on the surface of RRP12 helices 35–40 (Figure 6E). This is again consistent with the results of our *in vitro* binding assay and with our yeast two-hybrid analysis showing that yeast Slx9 interacts with the C-terminal repeat region of Rrp12 (Supplementary Figure S8D, E). In the human state RRP12-A, another region of SLX9 (residues 101–130) was in close contact with h1 of the 18S rRNA region, thus preventing integration of uS5. However, in contrast to the arrangement observed for human, in yeast this region was already occupied by uS5 in the early state Tsr1-1 (Figures 1, 6F).

In addition, two regions of RRP12 (residues 1070–1080 and 1145–1157) directly interact with eS17 on two opposite sides (Figure 6G). Furthermore, the very C terminus of RRP12 stretches through the 18S rRNA region near h28 and reaches from the solvent side to the inter-subunit side, where it interacts with the BUD23–TRMT112 complex (Figure 6H). Considering all these interactions, RRP12 is a central and integrating platform for coordinating AFs on the pre-40S head, including CK1, BYSL, LTV1, SLX9, BUD23 and TRMT112, thereby priming the pre-40S particle for nuclear export.

## DISCUSSION

In this study, we compared related nucleoplasmic pre-40S intermediates from two evolutionary distant organisms, human and yeast. The results give fundamental insight into the steps involved in 18S rRNA maturation, and point to a highly conserved pathway. According to the degree of rRNA maturation, ribosomal protein incorporation, AF occupancy, and mode of interaction, we can arrange these states in a common plausible and sequential order (Supplementary Figure S9). It is important to point out that the assembly steps may not all be strictly sequential as illustrated for example by the alternative entry points for uS5. Yet, although truly parallel assembly pathways may exist for the pre-40S, in particular under stress conditions, we did not observe intermediates in support of this notion. We therefore suggest that the main nucleoplasmic eukaryotic 40S ribosome maturation path has been visualized here, which represents a sequence of transitions from the primordial to the late cytoplasmic pre-40S ribosome. In the earliest yeast state—state Tsr1—the 40S head is largely delocalized and the immaturity of 18S rRNA h18 is maintained by Bud23. After the complete incorporation of TSR1 and the BUD23–TRMT112 complex, the pre-40S in humans reaches state UTP14. In the next state—state RRP12—additional factors are recruited and the 40S head and neck are further matured with the head domain now, for the first time, in a clearly defined position with respect to the main body of the 40S subunit. In this state, after the head has become an integral and structurally more rigid part of the pre-40S, a nuclear export-competent state is reached. After the successful maturation of 18S rRNA h18 and h34, Tsr1 is eventually released in state RIOK2-D2. In this scenario, maturation and integration of the 40S head turned out to be the major events that must occur in the nucleoplasm before the pre-40S is exported into the cytoplasm. This is in full agreement with previous biochemical observations establishing that the recruitment of ribosomal proteins that form the head is necessary for efficient pre-40S nuclear export in yeast as well as in human cells (65,66). Unfortunately, the high flexibility of the pre-40S head in all observed Tsr1 states did not allow for an analysis of the protein composition of the 40S head before state RRP12 (Supplementary Figure S9). Therefore, visualization of many incorporated 40S head proteins only in the later RRP12 states is very likely to be preceded by earlier recruitment events as described before for uS7 and uS9 (66,67).

There are several arguments supporting the notion that our structures (states Tsr1, UTP14, and RRP12) represent *bona fide* nucleoplasmic intermediates. First, our samples were purified under normal and not inhibited growth conditions using the nuclear export adapter protein SLX9, which has dual nucleolar and nucleoplasmic location (42,43). Second, the majority of BUD23 and TRMT112 has also been shown to localize to the nucleoplasm (24). Third, the 5' and central domains of the 18S rRNA showed an almost mature conformation resembling that of the primordial pre-40S, whereas the h18 of the 18S rRNA adopted a different immature conformation stabilized by the newly recruited Bud23. Fourth, compared to the primordial pre-40S, the 18S rRNA is further matured, including the 3' minor domain (helices h44 and h45) and formation of the CPK (14). Last, early 90S AFs (the Bms1 and Mpp10 modules, among others) are dissociated, whereas late pre-40S AFs (Rio1, Rio2, Nob1, etc.) have not yet been associated (or will be later).

Although state Tsr1-1 is very closely connected with the primordial pre-40S, we expect that there are still intermediates missing between these two steps. In this regard, we can consider the following points: (i) the RNA helicase Dhr1 is responsible for the release of U3 snoRNA from the 90S pre-ribosome (14,15,32,68). Interestingly, in the primordial pre-40S, it is in a nucleotide-free state, requiring ATP to start another round of catalysis (14). Thus, one obvious step might represent a Dhr1-driven remodeling event, in which the last remaining 90S factors dissociate (see accompanying paper by Cheng *et al.*). (ii) In 30S biogenesis by prokaryotes (such as *E. coli*), incorporation of h44 of the 16S RNA is highly regulated by the methyltransferase KsgA (69–71), a homolog of DIMT1. Thus, there might be a state that also shows this coordination. It is therefore possible that we may also have missed other intermediates in our preparation that still associate with TSR1 in state RIOK2-D and it is not clear that the dissociation has obligatorily to occur immediately after state RIOK2-D1. We speculate that there might be an intermediate flipped-out conformation of TSR1 present during the release, which was observed in the recruitment phase (state Tsr1-2).

Notably, the overall pre-40S assembly process in yeast and human cells is highly conserved. The folding sequence of the 18S rRNA and the corresponding AFs functioning in these individual steps essentially follow the same order with a few exceptions: (i) Although the resolution is insufficient to reveal the C-terminal helices of Slx9 in the yeast state Rrp12, it is clear that only in the human states UTP14 and RRP12-A does the N-terminal helix of SLX9 exist. (ii) As a result of the N-terminal helix of SLX9 associating tightly with the CPK, the ribosomal protein uS5 incorporates at different time points in yeast and human cells. In yeast, it can already interact with the CPK in the early state Tsr1-1 with uS2–eS21 entering later in the state Rrp12. However, in human cells, uS2–uS5–eS21 inserts into the particle as a protein cluster only in the late state RRP12-A2. 3) UTP14 remains associated with the 3' end of the 18S rRNA in the human state UTP14, however, in yeast it dissociates earlier in the Tsr1 state. 4) Besides UTP14, DIMT1 is another AF that dissociates at different time points. DIMT1 in human cells is a nuclear protein (24), whereas in yeast it migrates with the pre-40S ribosome from the nucleus into the cytoplasm. In the state post-A1 90S pre-ribosome, A1782 was inserted into its active pocket, clearly indicating early methylation at this site (14). However, it is also safe to assume that A1781 is also modified in the nucleus. Dim1 in the state Rio2 in yeast has a position-blocking role by competing with the C-terminal helix of Ltv1.

Even in light of these differences, overall, we observed an amazing similarity in the nucleoplasmic pre-40S formation between yeast and human, despite roughly 1 billion years of evolutionary distance. Thus, learning from yeast might help us to understand human diseases rooted in this segment of 40S biogenesis and *vice versa*, insights from the human system could promote fast and powerful yeast genetics that are not yet available in human.

## DATA AVAILABILITY

All cryo-EM maps and molecular models have been deposited in the Electron Microscopy Data Bank with accession IDs EMD-32792 [state Tsr1-1(uS5)], EMD-32793 [state Tsr1-1(no uS5)], EMD-32794 [state Tsr1-2(uS5)], EMD-32795 [state Tsr1-2(no uS5)], EMD-32796 (state Tsr1-3), EMD-32799 (state UTP14), EMD-32797 (state Rrp12-A), EMD-32800 [state RRP12-A1(CK1)], EMD-32801 [state RRP12-A1(no CK1)], EMD-32802 (state RRP12-A2), EMD-32803 (state RRP12-A3), EMD-32804 (state RRP12-B1), EMD-32806 (state RRP12-B2), EMD-32807 (state RRP12-B3), EMD-32798 (state Rio2-C), and EMD-32808 (state RIOK2-D2), and in the Protein Data Bank (PDB) with accession codes 7WTN [state Tsr1-1(uS5)], 7WTO [state Tsr1-1(no uS5)], 7WTP [state Tsr1-2(uS5)], 7WTQ [state Tsr1-2(no uS5)], 7WTR (state Tsr1-3), 7WTS (state UTP14), 7WTT [state RRP12-A1(CK1)], 7WTU [state RRP12-A1(no CK1)], 7WTV (state RRP12-A2), 7WTW (state RRP12-A3), 7WTX (state RRP12-B1), 7WTZ (state RRP12-B2), 7WU0 (state RRP12-B3).

## Supplementary Material

gkac961_Supplemental_File
