# Supplementary material for: The nucleoplasmic phase of pre-40S formation prior to nuclear export
Source: Nucleic Acids Res. 2022 Nov 2;50(20):11924–37. doi: 10.1093/nar/gkac961 (PMC9723619; doi:10.1093/nar/gkac961)
Supplement: gkac961_Supplemental_File [file gkac961_supplemental_file.pdf]

# The nucleoplasmic phase of pre-40S formation prior to nuclear export

Jingdong Cheng[1,2], Benjamin Lau[3], Mattias Thoms[1], Michael Ameismeier[1], Otto Berninghausen[1], Ed Hurt[3,*] and Roland Beckmann[1,*]

**Affiliations**

[1] Gene Center and Department of Biochemistry, University of Munich LMU, Feodor-Lynen-Str. 25, 81377 Munich, Germany.

[2] Institutes of Biomedical Sciences, Shanghai Key Laboratory of Medical Epigenetics, International Co-laboratory of Medical Epigenetics and Metabolism (Ministry of Science and Technology), Fudan University, Dong'an Road 131, 200032, Shanghai, China.

[3] BZH, University of Heidelberg, Im Neuenheimer Feld 328, 69120 Heidelberg, Germany

*Corresponding authors:

R. Beckmann, Email: beckmann@genzentrum.lmu.de;

E. Hurt, Email: ed.hurt@bzh.uni-heidelberg.de

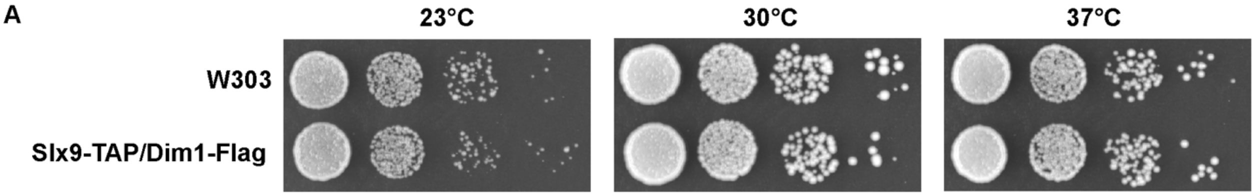


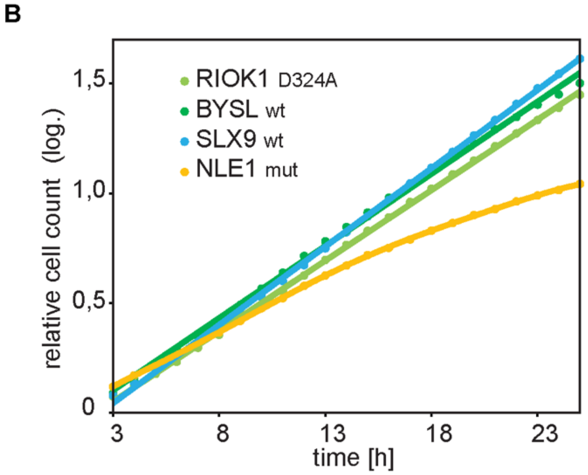


**Supplementary Figure S1. The growth analysis of the yeast strain and human cell lines.** **(A)** Growth analysis of yeast *Saccharomyces cerevisiae* strains with chromosomally integrated Slx9-TAP/Dim1-Flag in comparison to the W303 wild-type strain. Strains were grown on YPD plates at the indicated temperatures for 2 days. **(B)** The growth curves of cell lines used in this study with the human RIOK1, BYSL and SLX9 as C-terminally Flag-tagged baits inserted into HEK293/Flp-In cells compared to a cell line expressing a dominant human NLE1 mutant with a known growth defect. The time points refer to time after induction by tetracyclin with harvesting for preparation usually done 24 hours after induction. The calculated doubling time of the wild-type cell lines was between 14 and 15 hours. wt: wild-type gene with C-terminal Flag-tag inserted in HEK293/Flp-In cell line, mut: NLE1 mutant with C-terminal Flag-tag and disfunctional UBL-domain inserted in HEK293/Flp-In cell line.

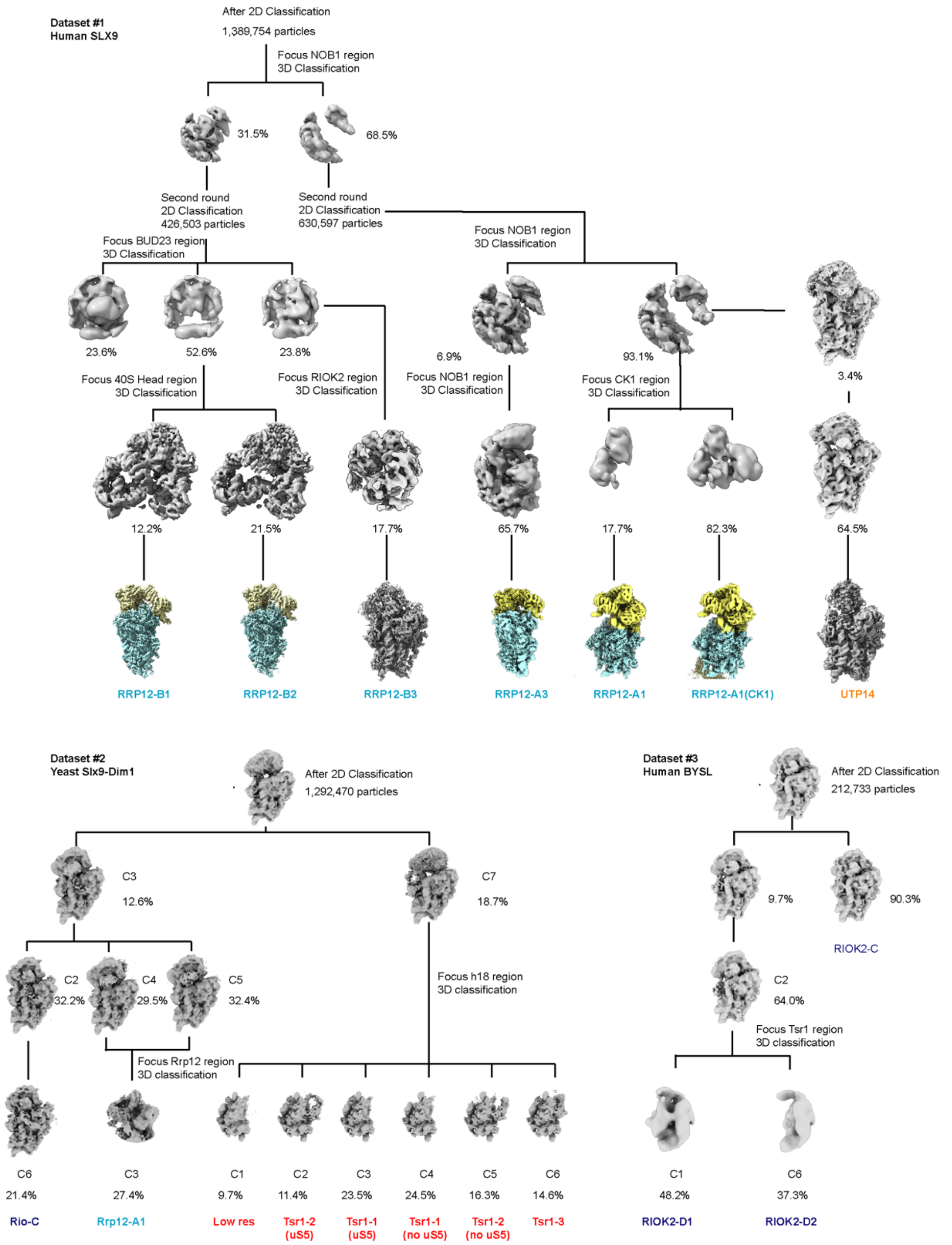


**Supplementary Figure S2. Sorting schemes of the human SLX9 and BYSL and yeast Slx9–Dim1 samples.**

Classification scheme of the three datasets used in this study. Datasets 1 and 3 were collected from human SLX9 and BYSL pull-outs, whereas dataset 2 was collected from yeast Slx9–Dim1 split-tag purification. After 3D classification, all classes resembling one of the states in this study are labeled accordingly. Classes that are not labeled either could not be further refined to higher resolution or could not be further classified despite the presence of large and highly flexible domains.

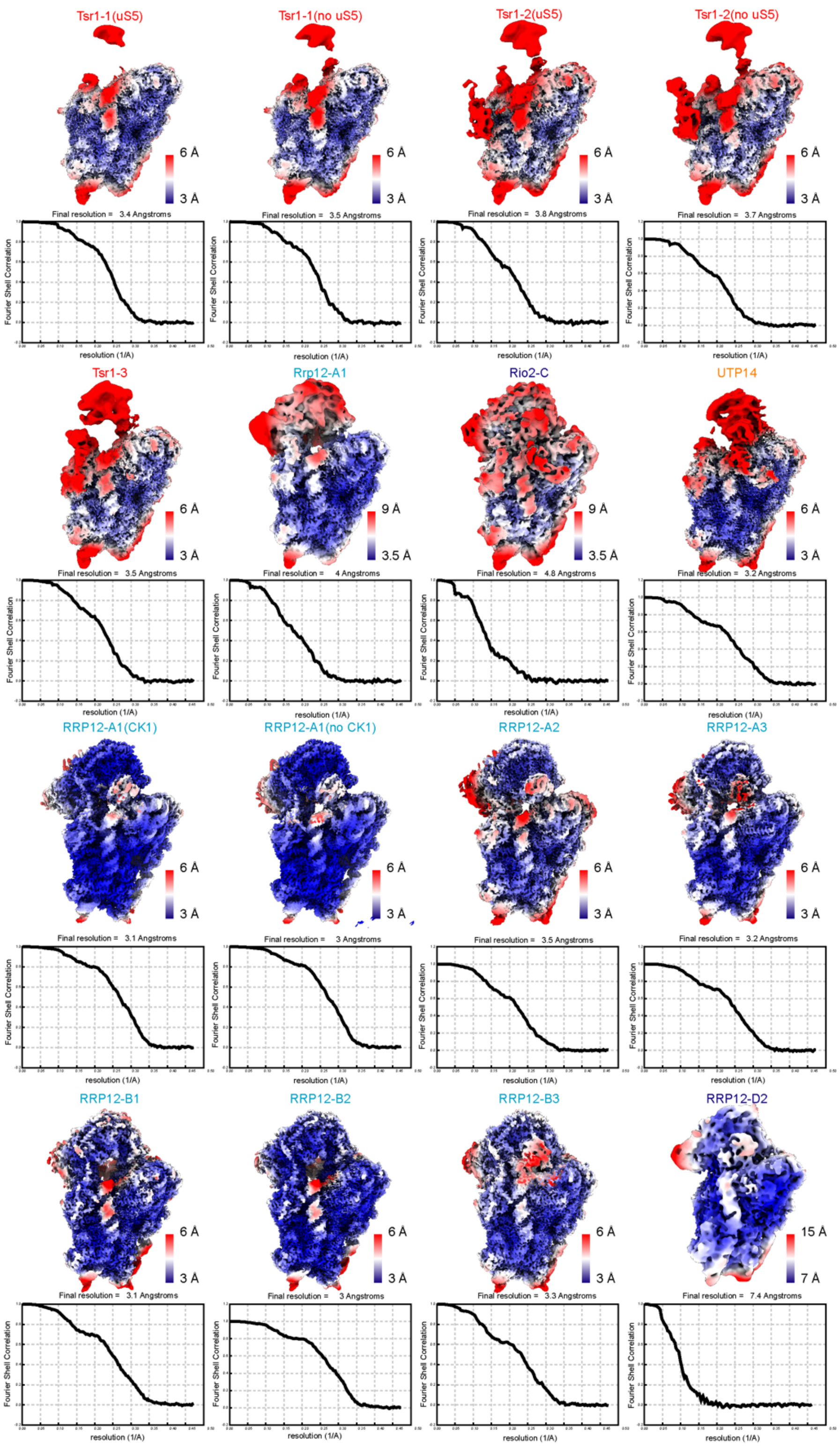


**Supplementary Figure S3. Local resolution and structural data validation.**
The local-resolution distributions of all the intermediates, as estimated by Relion (see individual blue-to-red scale bars). All states are shown in a 40S view. Shown below each structure are the gold-standard Fourier shell correlation curves that correspond to the reconstructions of all the states, as estimated by Relion.

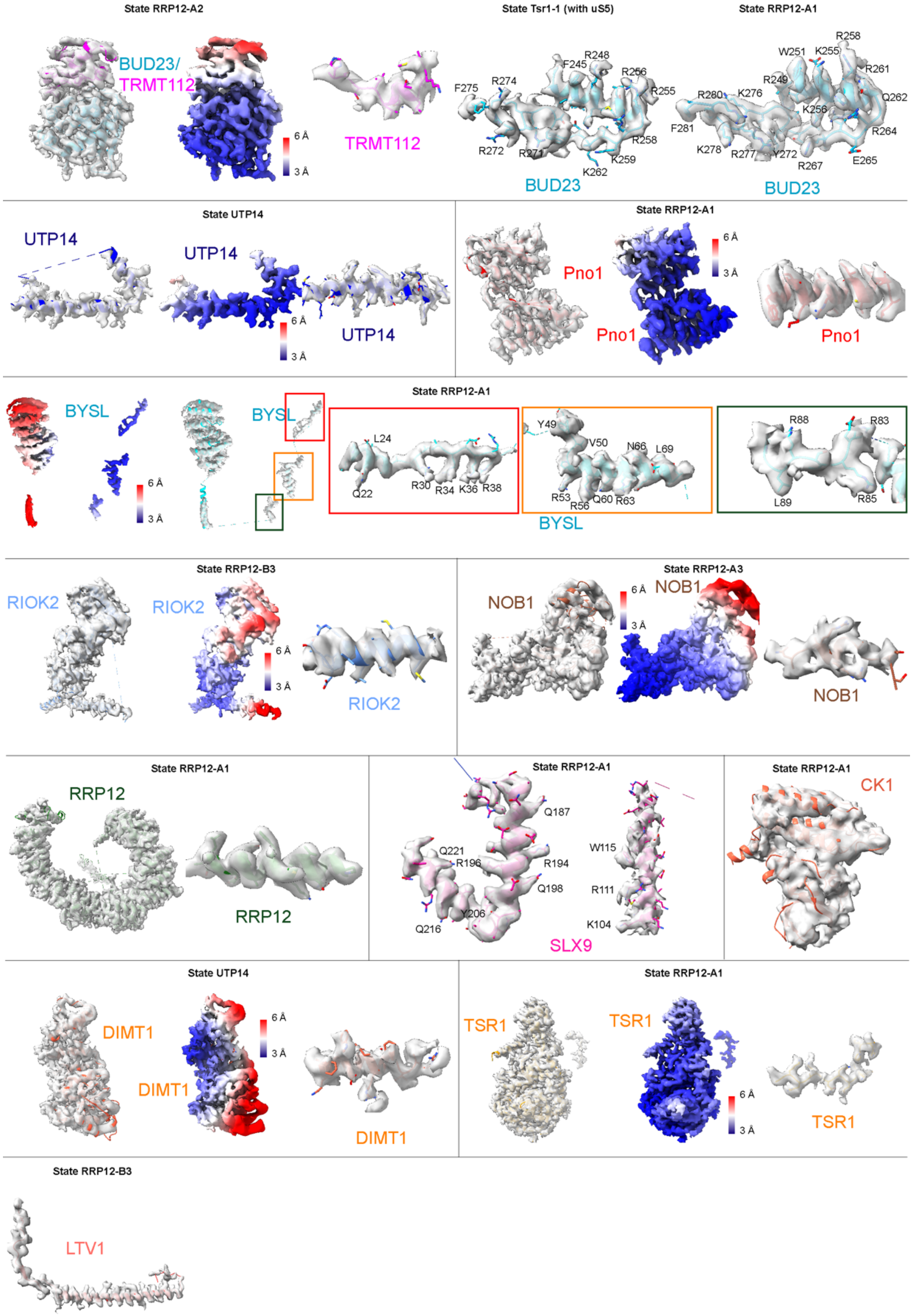


**Supplementary Figure S4. Structural validation of assembly factors**
Molecular models of the assembly factors discussed in this study and their cryo-EM density maps.

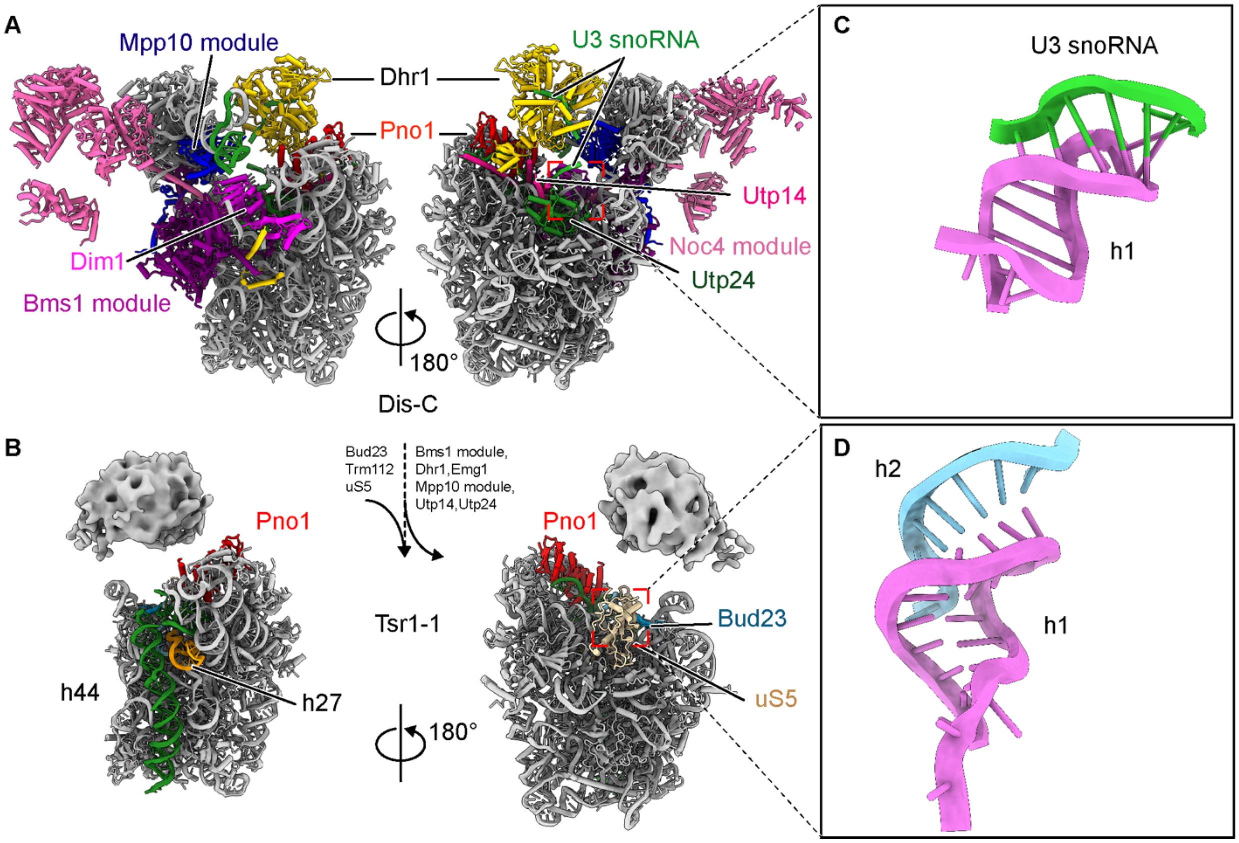


**Supplementary Figure S5. Comparison between states Dis-C and Tsr1-1.**
**(A, B)** Two views of molecular models for the yeast state Dis-C (**A**) and Tsr1-1 (with uS5, **B**). The highly flexible 40S head in state Tsr1-1 is indicated using the density map. Changes in composition are indicated in the middle. **(C, D)** The different conformations of h1 of the 18S rRNA region in yeast state Dis-C (**C**) and Tsr1-1 (**D**) to show the formation of the central pseudoknot (h2 of the 18S rRNA) in yeast state Tsr1-1.

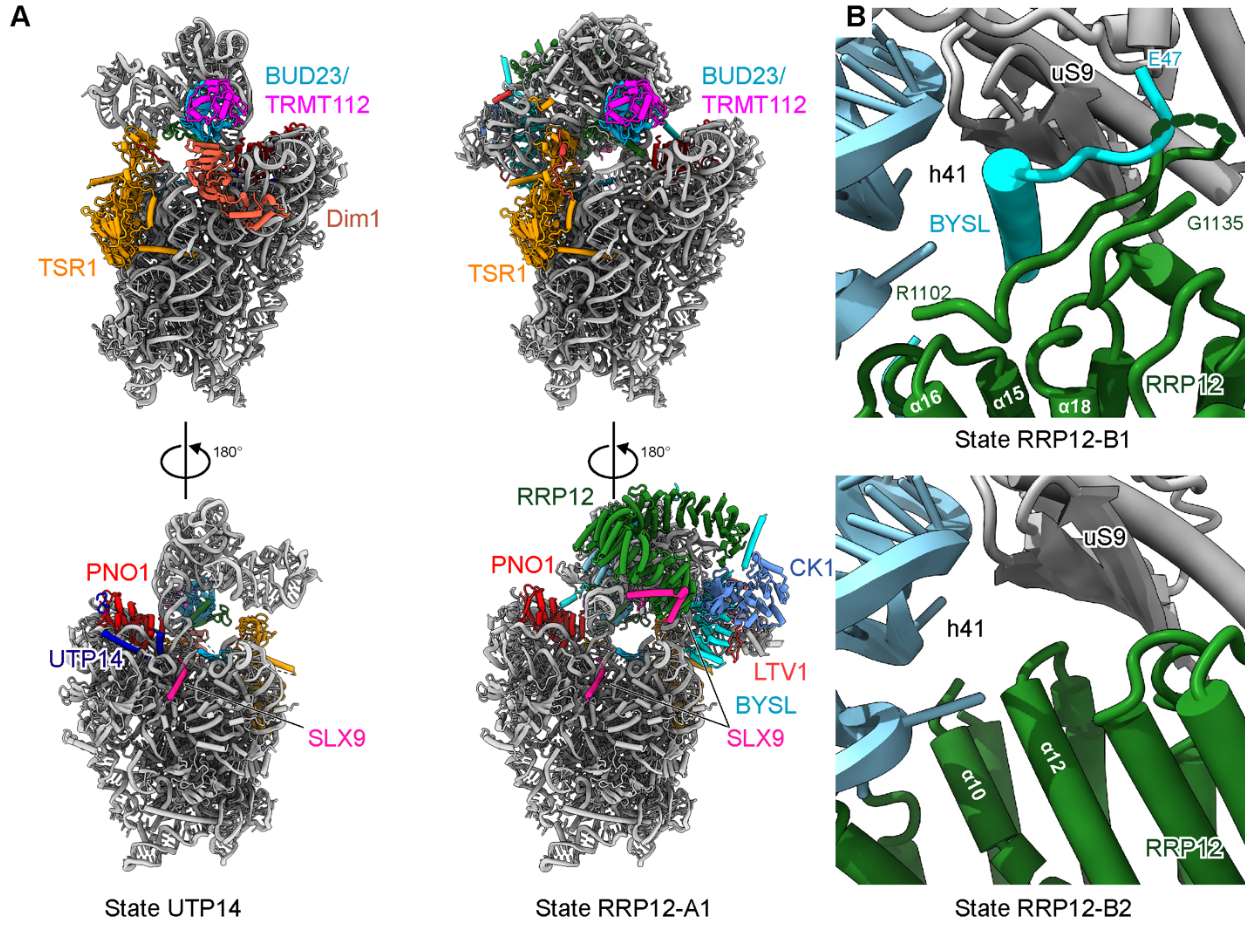


**Supplementary Figure S6. Molecular models of the human states UTP14 and RRP12-A1.** **(A)** Two views of the molecular models of the human states UTP14 (left) and RRP12-A1 (right); all assembly factors are indicated. **(B)** The N-terminal helix of BYSL pokes through a tunnel formed by h41 of the 18S rRNA, uS9, helices 15–18, and residues 1102–1135 of RRP12 in state RRP12-B1 (top). It is absent from the tunnel in state RRP12-B2 (bottom), allowing a sliding movement of the N-terminal ARM repeat of RRP12.

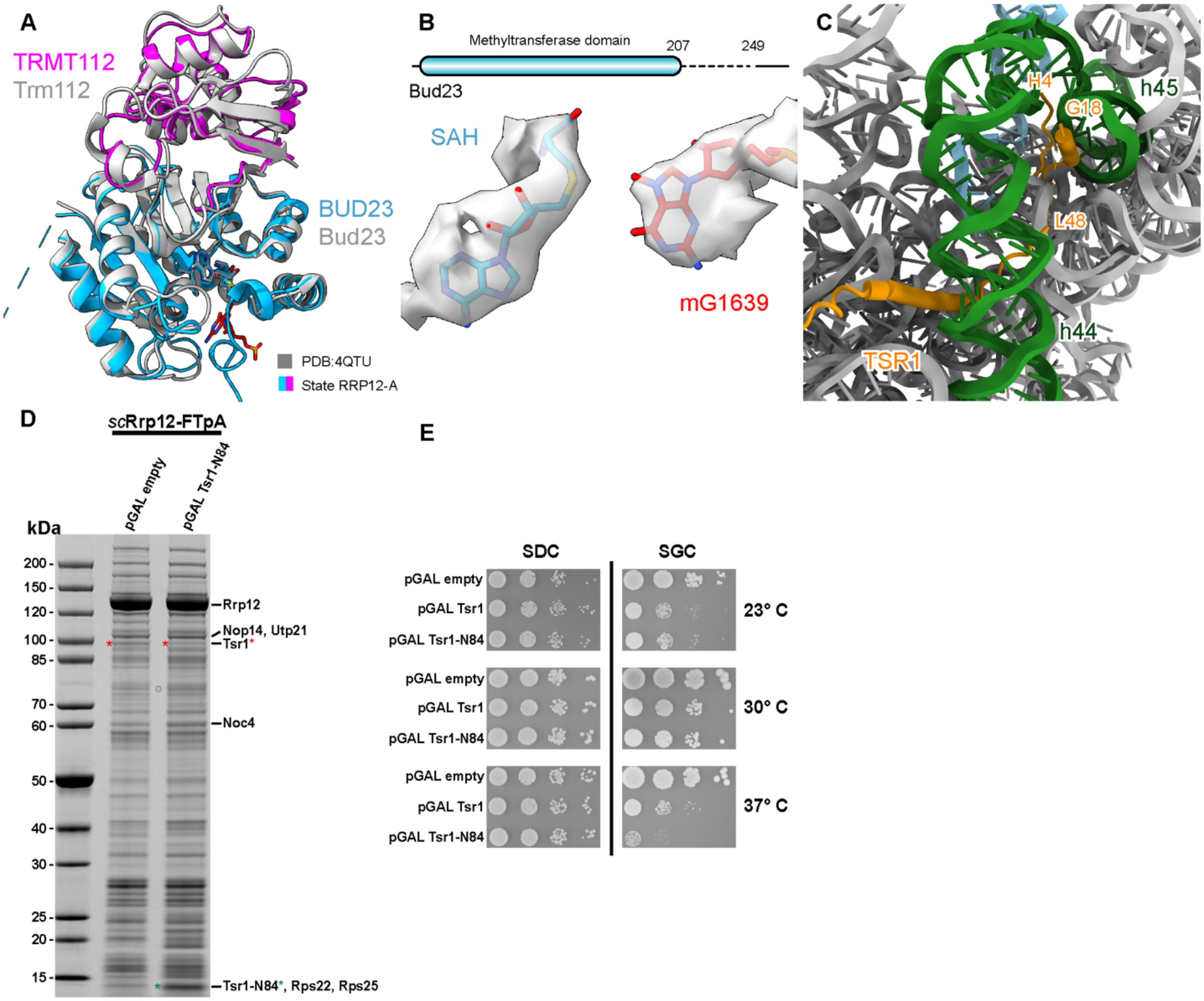


**Supplementary Figure S7. Methylation of G1639 and the interaction between the N terminus of Tsr1 and the pre-40S ribosome.**

**(A)** Structural comparision of the BUD23/TRMT112 complex from state RRP12-A with X-ray structure from yeast (PDB:4GTU). The X-ray structure was colored in gray. **(B)** SAH from BUD23 and m7G1639 of the 18S rRNA are shown in stick representation surrounded by density map. **(C)** The very N terminus of Tsr1 binds to a region deep within h28 of the 18S rRNA, and the N-terminal helix is inserted below h44. **(D)** Tandem affinity purification of *sc*Rrp12-FTpA from cells dependent on 6 h GAL-induced overexpression of plasmid-borne Tsr1 N terminus (N84) or empty vector. The final eluates were analyzed by SDS-PAGE and Coomassie staining, and the indicated bands were identified by mass spectrometry. **(E)** Growth analysis of GAL-induced plasmid-based Tsr1, Tsr1 N terminus (N84), and empty vector. Cells were spotted in 10-fold serial dilutions on SDC−LEU (left) or SGC−LEU (right) plates and growth was recorded after 2 days (SDC−LEU plates) or 4 days (SGC−LEU plates) at the indicated temperatures.

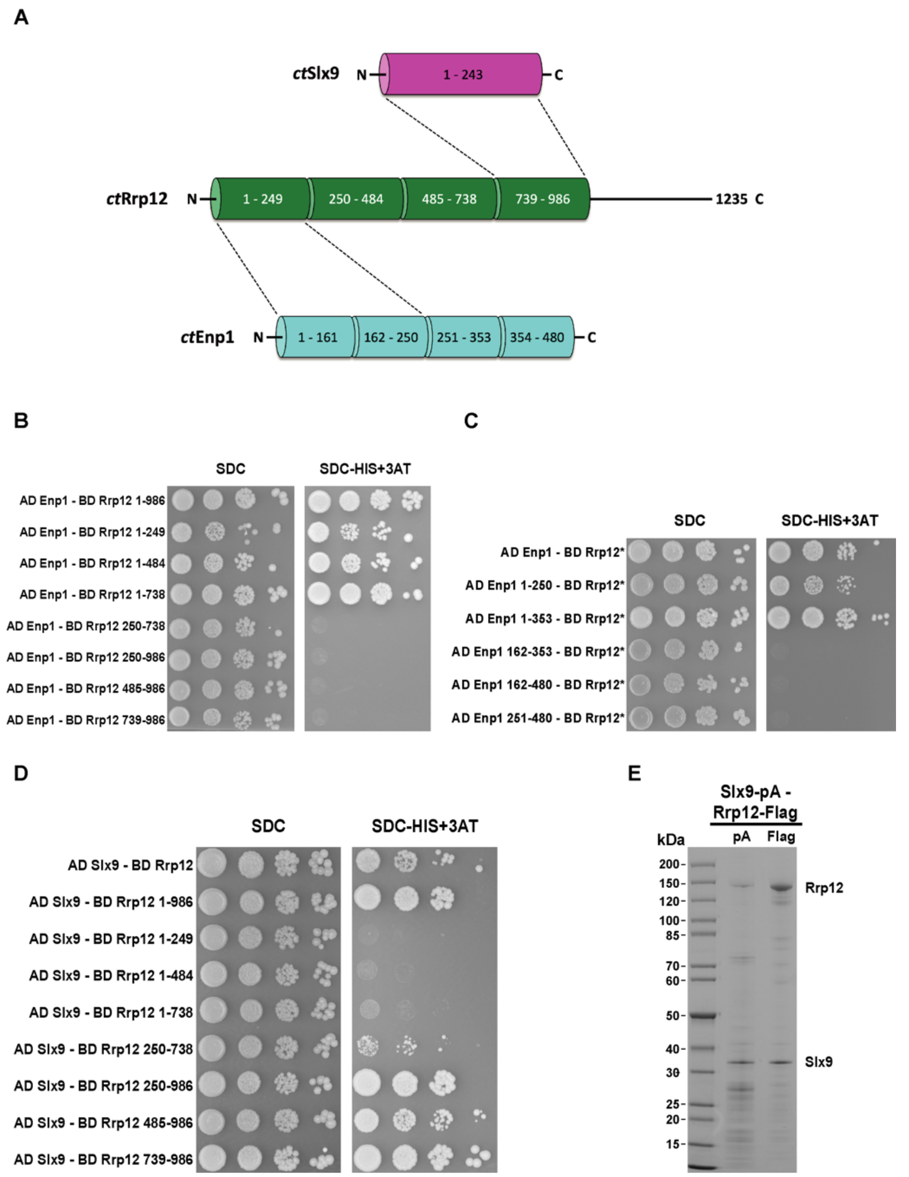


**Supplementary Figure S8. In vitro analysis of Rrp12 interacting with the assembly factors Enp1 and Slx9.**

**(A)** Schematic model of the analyzed Slx9 (magenta), Rrp12 (green), and Enp1 (cyan) constructs. Binding regions are indicated by dashed lines. **(B-D)** Yeast two-hybrid analysis of truncated *ct*Rrp12 and *ct*Enp1 binding to *ct*Slx9, *ct*Rrp12, or *ct*Enp1. The first protein was fused to the GAL4 activation domain (AD), whereas the second protein was fused to the GAL4 binding domain (BD). The yeast strain PJ69-4 was transformed with plasmids and 10-fold serial dilutions were spotted onto plates containing SDC−Trp−Leu (SDC) or SDC−Trp−Leu−His containing 2 mM 3AT (SDC−His+3AT) and growth was monitored after incubated for 4 days at 30 °C. For asterisk-labeled *ct*Rrp12, the truncated *ct*Rrp12ΔC (1–986 aa) construct was used owing to its superior biochemical properties. **(E)** Reconstitution of the *ct*Rrp12–Slx9 heterodimer. The tagged thermophilic Slx9-ProtA-TEV and Rrp12-Flag were overexpressed in Saccharomyces cerevisiae under the control of a GAL1-10 promoter. The TEV and Flag eluates from the tandem affinity purification were analyzed by SDS-PAGE and Coomassie staining.

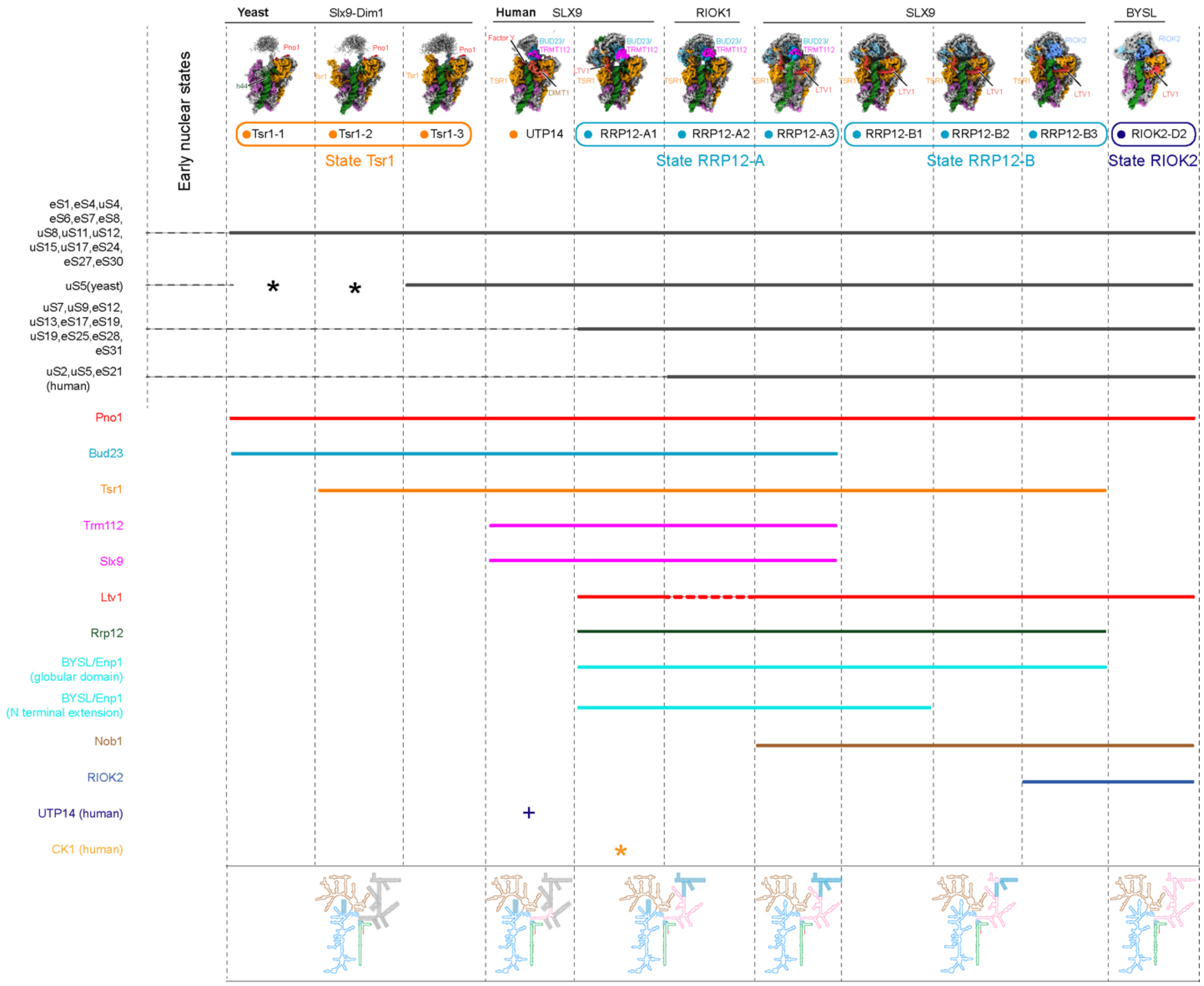


**Supplementary Figure S9. Schematic representation of AFs and rRNA secondary structure associated with all sequential states.**

Clustering and coloration are based on the time point of stable association and dissociation from the maturing particle as indicated by the horizontal lines. (*) indicating that factors in this state is/is not present. (+) indicating UTP14 is only present in state UTP14. Bold dashed lines for Ltv1 indicating that this factor was not visualized in this preparation of state RRP12-A2 after pulling with RIOK1 as bait, most likely due to dissocation during the preparation. Thin dashed lines for ribosomal proteins indicating that the recruitment of ribosomal proteins is likely to occur earlier (such as uS7 and uS9 on the 90S pre-ribosome, et al), however, due to high flexibility of the 40S head, they are not visable in the early pre-40S states. The invisible18S rRNA regions in the secondary structure are shadowed with light gray, while the immature regions are shadowed with light blue.

## Supplementary Table S1. Yeast strains used in this study.

| Name | Relevant genotype | Source |
|---|---|---|
| **PJ69-4A** | *MATα, trp1-901, leu2-3,112, ura3-52, his3-200, gal4Δ, gal80Δ, LYS2::GAL1-HIS3, GAL2-ADE2, met2::GAL7-lacZ* | James et al., 1996(*50*) |
| **W303** | *ade2-1, trp1-1, leu2-3,112, his3-11,15, ura3-1, can1-100* | Thomas and Rothstein, 1989(*49*) |
| **Slx9-TAP/Dim1-Flag** | *SLX9-TAP::HIS3, DIM1-Flag::natNT2, W303* | This study |
| **Rrp12-FTpA, YEplac112 empty** | *RRP12-TAP-Flag::natNT2, YEplac112 pGAL, W303* | This study |
| **Rrp12-FTpA, YEplac112 scTsr1 N84** | *RRP12-TAP-Flag::natNT2, YEplac112 pGAL scTSR1 N84, W303,* | This study |

## Supplementary Table S2. Cryo-EM data collection, refinement and validation statistics.

| | State Tsr1-1 (uS5) | State Tsr1-1 (no uS5) | State Tsr1-2 (uS5) | State Tsr1-2 (no uS5) | State Tsr1-3 | State Rrp12-A1 | State Rio2-C | State UTP14 | State RRP12-A1 (CK1) | State RRP12-A1 (no CK1) | State RRP12-A3 | State RRP12-B1 | State RRP12-B2 | State RRP12-B3 | State RRP12-A2 | State RIOK2-D2 |
|---|---|---|---|---|---|---|---|---|---|---|---|---|---|---|---|---|
| **Data collection and processing** | | | | | | | | | | | | | | | | |
| Magnification | | | | | | | | 47,214 | | | | | | | | 129,151 |
| Voltage (kV) | | | | | | | | | 300 | | | | | | | |
| Electron exposure (e–/Å$^2$) | | | | | | | | 44 | | | | | | | | 28 |
| Defocus range (μm) | | | | | | | | -0.8 to -2.5 | | | | | | | | -1 to -2.5 |
| Pixel size (Å) | | | | | | | | 1.059 | | | | | | | | 1.084 |
| Symmetry imposed | | | | | | | | | *C1* | | | | | | | |
| Initial particle images (no.) | | | | 1,292,470 | | | | | | | 1,389,754 | | | | 1,334,406 | 212,733 |
| Final particle images (no.) | 57,203 | 59,786 | 27,524 | 39,865 | 35,400 | 27,589 | 11,255 | 8,843 | 103,616 | 104,045 | 28,656 | 27,437 | 48,362 | 17,159 | 25,441 | 5,024 |
| Map resolution (Å) | 3.4 | 3.5 | 3.8 | 3.7 | 3.5 | 4 | 4.8 | 3.2 | 3.1 | 3 | 3.2 | 3.1 | 3 | 3.3 | 3.5 | 7.4 |
| FSC threshold | 0.143 | 0.143 | 0.143 | 0.143 | 0.143 | 0.143 | 0.143 | 0.143 | 0.143 | 0.143 | 0.143 | 0.143 | 0.143 | 0.143 | 0.143 | 0.143 |
| **Refinement** | | | | | | | | | Phenix.real_space_refine | | | | | | | |
| Model resolution (Å) | 3.3 | 3.3 | 3.6 | 3.6 | 3.3 | | | 3.1 | 3.3 | 3.2 | 3.2 | 3.1 | 3 | 3.3 | 3.5 | |
| FSC threshold | 0.5 | 0.5 | 0.5 | 0.5 | 0.5 | | | 0.5 | 0.5 | 0.5 | 0.5 | 0.5 | 0.5 | 0.5 | 0.5 | |
| Map sharpening *B* factor (Å$^2$) | -90 | -86 | -84 | -88 | -80 | | | -55 | -78 | -78 | -67 | -65 | -64 | -55 | -39 | |
| Model composition | | | | | | | | | | | | | | | | |
| Non-hydrogen atoms | 46,427 | 44,490 | 52,944 | 52,944 | 53,512 | | | 62,068 | 83,685 | 81,223 | 87,404 | 85,252 | 85,114 | 87,712 | 84,213 | |
| Protein residues | | | | | | | | | | | | | | | | |
| RNA | 2,635 | 2,383 | 3,296 | 3,296 | 3,259 | | | 3,787 | 6,255 | 5,958 | 6,720 | 6,393 | 6,331 | 6,656 | 6,355 | |
| Ligands | 1,200 | 1,200 | 1,255 | 1,255 | 1,298 | | | 1,482 | 1,575 | 1,575 | 1,587 | 1,605 | 1,624 | 1,624 | 1,575 | |
| | 2 | 2 | 2 | 2 | 2 | | | 0 | 2 | 2 | 4 | 4 | 4 | 4 | 2 | |
| *B* factors (Å$^2$) | 63.55 | 84.82 | 101.5 | 98.16 | 90.36 | | | 41.21 | 14.85 | 15.04 | 21.10 | 18.35 | 26.32 | 36.25 | 32.96 | |
| Protein | 52.40 | 69.82 | 2 | 89.23 | 79.11 | | | 128.1 | 13.47 | 13.69 | 21.23 | 15.18 | 19.24 | 33.23 | 32.58 | |
| RNA | 72.62 | 95.90 | 90.29 | 106.9 | 100.8 | | | 7 | 16.91 | 16.96 | 20.90 | 23.08 | 36.63 | 40.85 | 33.53 | |
| Ligand | 112.4 | 127.6 | 112.5 | 129.4 | 6 | | | 53.75 | 23.89 | 24.81 | 27.08 | 27.49 | 49.96 | 54.33 | 57.67 | |
| | 8 | 0 | 1 | 7 | 161.9 | | | 0 | | | | | | | | |
| | | | 185.0 | | 3 | | | | | | | | | | | |
| | | | 8 | | | | | | | | | | | | | |
| R.m.s. deviations | | | | | | | | | | | | | | | | |
| Bond lengths (Å) | 0.005 | 0.005 | 0.007 | 0.005 | 0.006 | | | 0.014 | 0.006 | 0.005 | 0.005 | 0.008 | 0.004 | 0.003 | 0.006 | |
| Bond angles (°) | 0.820 | 0.826 | 0.951 | 0.879 | 0.868 | | | 0.885 | 0.894 | 0.863 | 0.845 | 1.000 | 0.830 | 0.781 | 0.902 | |
| Validation | | | | | | | | | | | | | | | | |
| MolProbity score | 1.81 | 1.83 | 1.96 | 1.87 | 1.81 | | | 1.58 | 1.61 | 1.62 | 1.66 | 1.62 | 1.55 | 1.59 | 1.65 | |
| Clashscore | 5.74 | 6.32 | 8.18 | 6.91 | 6.31 | | | 5.73 | 6.11 | 5.90 | 6.56 | 6.10 | 5.17 | 5.87 | 6.22 | |
| Poor rotamers (%) | 0.09 | 0.05 | 0.04 | 0.04 | 0 | | | 0.06 | 0 | 0.02 | 0.03 | 0.02 | 0.02 | 0.05 | 0.04 | |
| Ramachandran plot | | | | | | | | | | | | | | | | |
| Favored (%) | 91.65 | 92.05 | 91.14 | 92.04 | 92.69 | | | 96.08 | 95.97 | 95.79 | 95.68 | 95.87 | 95.98 | 96.03 | 95.64 | |
| Allowed (%) | 8.27 | 7.87 | 8.73 | 7.84 | 7.22 | | | 3.89 | 3.99 | 4.20 | 4.28 | 4.04 | 3.94 | 3.89 | 4.34 | |
| Disallowed (%) | 0.08 | 0.09 | 0.12 | 0.12 | 0.09 | | | 0.03 | 0.03 | 0.02 | 0.05 | 0.10 | 0.08 | 0.08 | 0.02 | |
| **EMDB** | 32792 | 32793 | 32794 | 32795 | 32796 | 32797 | 32798 | 32799 | 32800 | 32801 | 32803 | 32804 | 32806 | 32807 | 32802 | 32808 |
| **PDB** | 7WTN | 7WTO | 7WTP | 7WTQ | 7WTR | | | 7WTS | 7WTT | 7WTU | 7WTW | 7WTX | 7WTZ | 7WU0 | 7WTV | |

**Supplementary Table S3. Molecular models of the nucleaplasmic pre-40S ribosome.**

| Assembly factors | Full length (aa) | Modeled Regions (aa) | Modeling | Present in States |
|---|---|---|---|---|
| **Yeast** | | | | |
| Bud23 | 275 | 241-279 | *de nove* build | Tsr1 |
| Dim2/Pno1 | 274 | 93-273 | *de nove* build | All |
| Tsr1 | 788 | 13-33 | *de nove* build | Tsr1-2, Tsr1-3 |
| | | 43-310, 335-355, 484-787 | From PDB:6EML | Tsr1-2, Tsr1-3 |
| **Human** | | | | |
| BUD23/ WBSCR22 | 281 | 6-207, 249-281 | *de nove* build | UTP14, RRP12-A |
| DIMT1 | 313 | 37-313 | From PDB: 1ZQ9 | UTP14 |
| PNO1 | 252 | 73-247 | *de nove* build | All |
| BYSL | 437 | 18-39, 47-72, 83-91, 142-167 | *de nove* build | RRP12, RIOK2 |
| | | 180-427 | From PDB:6G18 | |
| CK1 | 415 | 9-305 | From PDB: 6GZM | RRP12-A1(CK1) |
| LTV1 | 475 | 247-270 | From PDB:6G18 | RRP12, RIOK2 |
| | | 291-314, 352-369, 414-474 | *de nove* build | |
| NOB1 | 412 | 7-131, 206-222, 230-412 | From PDB:6G18 | RRP12-A3, RRP12-B, RIOK2 |
| RIOK2 | 552 | 9-299, 494-523, 526-544 | From PDB:6G18 | RRP12-B3, RIOK2 |
| RRP12 | 1297 | 106-224, 232-266, 278-295, 301-701, 708-1043, 1070-1080, 1102-1111, 1115-1135, 1145-1157, 1188-1216, 1229-1257 | *de nove* build | RRP12 |
| SLX9/ C21orf70 | 230 | 101-130, 174-225 | *de nove* build | UTP14, RRP12-A1, RRP12-A3 |
| TRMT112 | 125 | 1-118 | *de nove* build | UTP14, RRP12-A |
| TSR1 | 804 | 4-18, 48-104, 109-313, 342-363, 392-405, 464-792 | *de nove* build | UTP14, RRP12, RIOK2-C, RIOK2-D1 |
| UTP14 | 771 | 220-263，627-640 | *de nove* build | UTP14 |

**Supplementary Table S4. Nomenclature of orthologous yeast and human assembly factors.**

| Yeast name | Human name |
|---|---|
| Bud23 | BUD23/ WBSCR22 |
| Dim1 | DIMT1 |
| Dim2/Pno1 | PNO1 |
| Enp1 | BYSL |
| Hrr25/Ck1 | CK1 |
| Ltv1 | LTV1 |
| Nob1 | NOB1 |
| Rio2 | RIOK2 |
| Rrp12 | RRP12 |
| Slx9 | SLX9/ C21orf70 |
| Trm112 | TRMT112 |
| Tsr1 | TSR1 |
| Utp14 | UTP14 |
